# The Major RNA-Binding Protein ProQ Impacts Virulence Gene Expression in Salmonella enterica Serovar Typhimurium

**DOI:** 10.1128/mBio.02504-18

**Published:** 2019-01-02

**Authors:** Alexander J. Westermann, Elisa Venturini, Mikael E. Sellin, Konrad U. Förstner, Wolf-Dietrich Hardt, Jörg Vogel

**Affiliations:** a Helmholtz Institute for RNA-based Infection Research (HIRI), Würzburg, Germany; b Institute of Molecular Infection Biology (IMIB), University of Würzburg, Würzburg, Germany; c Core Unit Systems Medicine, University of Würzburg, Würzburg, Germany; d Institute of Microbiology, ETH Zürich, Zürich, Switzerland; The Sanger Institute; Centre International de Recherche en Infectiologie; University of Liverpool

**Keywords:** Hfq, noncoding RNA, ProQ, RNA-seq, bacterial pathogen, posttranscriptional control

## Abstract

FinO domain proteins such as ProQ of the model pathogen Salmonella enterica have emerged as a new class of major RNA-binding proteins in bacteria. ProQ has been shown to target hundreds of transcripts, including mRNAs from many virulence regions, but its role, if any, in bacterial pathogenesis has not been studied. Here, using a Dual RNA-seq approach to profile ProQ-dependent gene expression changes as *Salmonella* infects human cells, we reveal dysregulation of bacterial motility, chemotaxis, and virulence genes which is accompanied by altered MAPK (mitogen-activated protein kinase) signaling in the host. Comparison with the other major RNA chaperone in *Salmonella*, Hfq, reinforces the notion that these two global RNA-binding proteins work in parallel to ensure full virulence. Of newly discovered infection-associated ProQ-bound small noncoding RNAs (sRNAs), we show that the 3′UTR-derived sRNA STnc540 is capable of repressing an infection-induced magnesium transporter mRNA in a ProQ-dependent manner. Together, this comprehensive study uncovers the relevance of ProQ for *Salmonella* pathogenesis and highlights the importance of RNA-binding proteins in regulating bacterial virulence programs.

## Abstract (precis)

The protein ProQ has recently been discovered as the centerpiece of a previously overlooked “third domain” of small RNA-mediated control of gene expression in bacteria. As *in vitro* work continues to reveal molecular mechanisms, it is also important to understand how ProQ affects the life cycle of bacterial pathogens as these pathogens infect eukaryotic cells. Here, we have determined how ProQ shapes *Salmonella* virulence and how the activities of this RNA-binding protein compare with those of Hfq, another central protein in RNA-based gene regulation in this and other bacteria. To this end, we apply global transcriptomics of pathogen and host cells during infection. In doing so, we reveal ProQ-dependent transcript changes in key virulence and host immune pathways. Moreover, we differentiate the roles of ProQ from those of Hfq during infection, for both coding and noncoding transcripts, and provide an important resource for those interested in ProQ-dependent small RNAs in enteric bacteria.

## INTRODUCTION

RNA-binding proteins (RBPs) are at the heart of central cellular processes in all living organisms. In bacteria, their functions range from being structural components of essential ribonucleoprotein complexes, such as the ribosome, to facilitate posttranscriptional control of mRNAs, often in conjunction with small regulatory RNAs (sRNAs) (1). RBPs have also increasingly been found to play roles in regulating virulence and stress response genes, helping to ensure the full infectivity and survival of bacterial pathogens inside their host.

Salmonella enterica serovar Typhimurium (henceforth *Salmonella*) has served as an important model for studying the functions of virulence-associated RBPs. This major Gram-negative bacterium is closely related to Escherichia coli and infects both humans and livestock. *Salmonella* virulence is predominantly mediated by effector proteins that are translocated into eukaryotic host cells via two type III secretion systems (T3SSs) encoded on the major *Salmonella* pathogenicity islands (SPIs): SPI-1, required for host cell invasion (2), and SPI-2, for intracellular survival (3). Other SPIs as well as *Salmonella* motility and chemotaxis loci also contribute, for example, by enabling the bacteria to sense and reach the proper host niche (4). Importantly, these infection-relevant genes are not only controlled transcriptionally by various DNA-binding proteins, they are also targeted at the RNA level by the global RBPs Hfq, CsrA, and CspC/E, each of which interacts with hundreds of different *Salmonella* transcripts (5–8). Furthermore, Hfq, CsrA, and CspC/E are essential for *Salmonella* virulence in mouse models of infection (6, 9, 10).

To date, most efforts to understand RBP functions in *Salmonella* have focused on Hfq, an RNA chaperone that facilitates the base pairing of ∼100 different sRNAs with target mRNAs (11, 12). Early reports that *hfq* deletion impairs many virulence-related functions (9) motivated mechanistic studies on numerous sRNAs (from both the *Salmonella* core genome and the SPIs) that directly repress or activate mRNAs of infection-relevant genes. However, the observation that many sRNAs are Hfq independent (5, 7) hinted at the existence of another sRNA-related RBP in enteric pathogens. Indeed, the protein ProQ was recently reported to be a previously unrecognized global RBP in *Salmonella* and E. coli (13).

ProQ is a ∼25-kDa protein that was originally noted in E. coli for its positive effect on the cellular levels of ProP (a proline transporter) and thus osmolyte accumulation (14, 15). The first hints of RNA-binding activity came from similarities in the protein sequence to FinO, a plasmid-encoded RBP required for *cis*-antisense RNA-mediated control of conjugation (16). Proteins containing ProQ/FinO domain(s) are present in many alpha-, beta-, and gammaproteobacteria on both chromosomes and mobile elements (13, 17–19). Biochemical and structural analyses of E. coli ProQ have fully demonstrated its ability to bind RNA *in vitro* (20, 21). More importantly, profiling of ProQ ligands in *Salmonella* identified an unexpectedly large suite of RNA targets, which includes both hundreds of mRNAs and dozens of sRNAs (13, 22). Two molecular functions for ProQ have been established. First, translational mRNA repression by a *trans*-encoded sRNA that requires ProQ for its function (23), a mechanism resembling that of the FinO-like protein RocC in sRNA-mediated competence regulation in Legionella pneumophila (24). Second, ProQ stabilizes mRNAs by binding at their 3′ ends, protecting them against decay by exonuclease RNase II (22).

Both Hfq and ProQ have been found to target many mRNAs from pathogenicity loci (5, 7, 9, 13, 22). Therefore, as *in vitro* work continues to reveal molecular mechanisms, it is also important to understand how ProQ affects the most important phase of the life cycle of *Salmonella* as a pathogen, i.e., the infection of eukaryotic cells. However, in contrast to the established central role of Hfq in *Salmonella* virulence (25), the *proQ* gene was not disrupted in previous transposon-based virulence screens in various animal models of salmonellosis (26, 27), precluding an assessment of the importance of ProQ for pathogenesis. Consequently, we sought to determine whether and how ProQ shapes *Salmonella* virulence and how the activities of these two major RBPs compare with each other in the infection process.

In this study, we apply global transcriptomics of pathogen and host cells during infection. In doing so, we reveal ProQ-dependent transcript changes in key virulence and host immune pathways. Moreover, we differentiate the roles of ProQ from those of Hfq during infection for both coding and noncoding transcripts.

## RESULTS

### Attenuated virulence of a *Salmonella* Δ*proQ* mutant in cultured HeLa cells.

To identify a cell culture-based model to test for a putative role of ProQ in *Salmonella* virulence, we moved the previously described *proQ* deletion (13) into a *Salmonella* strain that constitutively expresses GFP (28) and infected several established host cell types for the study of *Salmonella* pathogenesis (see Fig. S1 and S2 in the supplemental material). This strain (Δ*proQ*/*gfp*^+^) was complemented using plasmid pZE12-ProQ (13) which yields a mild (∼2- to 3-fold) overexpression of ProQ (Fig. S1b). Invasion and intracellular replication rates were quantified using flow cytometry to measure the bacterial GFP signal inside host cells. Of the three cell types tested, *Salmonella* Δ*proQ* bacteria exhibited a ∼2-fold reduced invasion compared to the wild-type strain into HeLa cells (Fig. S1d). This invasion defect was only partially restored in the complemented strain, suggesting that ProQ levels must be tightly controlled for successful host invasion. In contrast, ProQ deficiency or overexpression only led to a subtle decrease in intracellular replication (Fig. S1e). When testing human phagocytic cells, we found only mild differences between the above mutant strains with respect to uptake or intracellular replication in human monocytic or macrophage-like THP-1 cells (Fig. S2a and b).

10.1128/mBio.02504-18.1 FIG S1 Virulence parameters of *Salmonella proQ* mutants in HeLa cell culture assays. (a) *proQ* deletion strategy. The 5′ portion of *proQ* (including its own promoter; red arrow) is deleted from the *Salmonella* genome, while the 3′ portion is kept as it contains the promoter of the downstream gene *prc* (as deduced from the respective site in the E. coli genome [85]; blue arrow with the asterisk). The remaining TSS annotations were obtained from reference 64 (black arrows). The read coverage plots show RNA-seq data for two biological replicates of wild-type or Δ*proQ Salmonella* and confirm the deletion of the respective genomic region. Note that the downstream *prc* gene is slightly induced in the *proQ* deletion background. (b) Wild-type *Salmonella* harboring an empty control vector, a Δ*proQ* strain with the same empty vector, or a Δ*proQ* strain overexpressing *proQ* from that plasmid (strain *proQ*++) in either the wild-type background (GFP-negative; black) or P*_tet_::gfp* background (green) were grown under SPI-1- or SPI-2-inducing conditions (see Materials and Methods), and the levels of ProQ protein were determined by Western blotting using an antibody against the endogenous protein. GroEL served as a loading control. (c) Assessment of the risk of plasmid loss during infection. HeLa cells were infected with wild-type *Salmonella* containing an ampicillin resistance-mediating plasmid (MOI of 10) in the presence or absence of ampicillin in the infection medium, and after 24 h, host cells were lysed, and intracellular bacteria were recovered and plated in parallel on LB only or LB-Amp plates. (d) *proQ* deletion causes a ∼2-fold reduction in invasion of *Salmonella* into HeLa cells. *Salmonella* input suspensions were derived from bacteria in late exponential phase (OD_600_ of 2.0). The fraction of infected cells at 0 h and 24 h p.i. is shown. Significance was evaluated using a two-tailed Student’s *t* test, and *P* values of <0.05 were considered statistically significant and are denoted by an asterisk (*). *P* values of <0.01 were considered very significant (**), and *P* values of <0.001 were considered extremely significant (***). n.s., not significant. (e) Intracellular replication inside HeLa cells is not affected by *proQ* deletion or overexpression. In panels d and e, HeLa cells were infected with the indicated *Salmonella* strains (GFP-expressing background) at an MOI of 5. The proportion of invaded (GFP-positive) cells and the rate of intracellular replication (as deduced from the increase in GFP signal intensity per infected HeLa cell over time) were determined by flow cytometry. Data in panels c to e represent the means ± SD from at least three biological replicate experiments. Download FIG S1, PDF file, 0.1 MB. Copyright © 2019 Westermann et al. 2019 Westermann et al. This content is distributed under the terms of the Creative Commons Attribution 4.0 International license.

10.1128/mBio.02504-18.2 FIG S2 Assessment of an effect of ProQ on *Salmonella* virulence in monocyte and macrophage models. (a) Rate of infected (GFP-positive) THP-1 cells at 30 min and 24 h p.i. (MOI of 10) for the indicated *Salmonella* strains. THP-1 cells were either nondifferentiated (monocytes) or differentiated *in vitro* by adding phorbol 12-myristate 13-acetate (PMA) into the cell medium 3 days prior to infection (macrophages). *Salmonella* input suspensions were either derived from exponentially growing cultures (OD_600_ of 2.0) (exponential) or from overnight cultures opsonized for 20 min in mouse serum (stationary). Significance was evaluated using a two-tailed Student’s *t* test, and *P* values of <0.05 were considered statistically significant and are denoted by an asterisk. n.s., not significant. In differentiated THP-1 cells, none of the ProQ-dependent changes were statistically significant. (b) Fold replication of the same strains in the same host cell types between 30 min and 24 h p.i. (MOI of 10). Panels a and b report flow cytometry data (mean ± SD) from at least three biological replicate experiments. Note that for fully differentiated THP-1 cells (but not monocytic THP-1), the rate of *Salmonella*-containing cells increased over time, because macrophages but not monocytic cells engulf bacteria released from neighboring cells. (c) THP-1 cell death after infection with the indicated *Salmonella* strains (MOI of 10). Cell death was determined by measuring the rate of lactate dehydrogenase (LDH) released into the culture supernatant at 24 h p.i. The data indicate the means ± SD from at least three biological replicates. Download FIG S2, PDF file, 0.04 MB. Copyright © 2019 Westermann et al. 2019 Westermann et al. This content is distributed under the terms of the Creative Commons Attribution 4.0 International license.

### ProQ effects on host gene expression in *Salmonella*-infected HeLa cells.

Because of the rather subtle phenotypes in infection experiments of cultured cells, we employed the more sensitive Dual RNA-seq technique (29) to assess molecular consequences of ProQ deficiency, using the transcriptomes of the pathogen and host as a readout. This approach has been successfully used in the past to extract molecular phenotypes of, for example, infection-induced sRNAs in the absence of strong macroscopic phenotypes (30). In the present study, we selected HeLa cells to determine ProQ-dependent gene expression changes during the course of *Salmonella* infection. Following infection with *Salmonella* wild-type or Δ*proQ* bacteria, infected HeLa cells (GFP-positive) were enriched at 8 h and 16 h postinfection (p.i.), and subjected to Dual RNA-seq (Fig. S3a and b; Table S1A).

10.1128/mBio.02504-18.3 FIG S3 Experimental overview of the Dual RNA-seq screen for ProQ-dependent expression changes during *Salmonella* infection of HeLa cells. (a) Dual RNA-seq pipeline. HeLa cells were infected with wild-type or Δ*proQ Salmonella* (GFP-expressing strains), and invaded (GFP-positive) cells were enriched by FACS. Total RNA was extracted, and ribosomal transcripts of host and pathogen were depleted prior to sequencing. (b) Comparative Dual RNA-seq outline. RNA samples were taken before infection (0 h; lysate from *Salmonella* in exponential growth phase after the medium shift from LB to host cell media, artificially mixed with HeLa lysate prior to joint RNA extraction) or at 8 and 16 h after infection (mixed human-*Salmonella* infection samples). Each two biological replicates were analyzed. (c) Dual RNA-seq mapping statistics. Of the ∼20 million reads per sample that could be aligned to the reference annotations, between 1% (0 h and 8 h) and 2% (16 h) mapped to *Salmonella* and the remaining 98 or 99% mapped to the human genome. (d) RNA class distribution of reads mapped to the *Salmonella* genome. (e) RNA class distribution of reads mapped to the human genome. Abbreviations in panels d and e: rRNA, ribosomal RNA; tRNA, transfer RNA; mRNA, messenger RNA; sRNA, bacterial small regulatory RNA; IGRs, intergenic regions; lncRNA, long noncoding RNA; miRNA, microRNA; sn(o)RNA, small nucle(ol)ar RNA; miscRNA, miscellaneous RNA. The percentage values denote the range of the fraction of reads mapping to the corresponding RNA class (relative to the total fraction of reads assigned to the respective organism) for the two replicate experiments. Download FIG S3, PDF file, 0.1 MB. Copyright © 2019 Westermann et al. 2019 Westermann et al. This content is distributed under the terms of the Creative Commons Attribution 4.0 International license.

10.1128/mBio.02504-18.10 TABLE S1 Compiled data sets of the present study. Access table for detailed descriptions. Download Table S1, XLSX file, 23.0 MB. Copyright © 2019 Westermann et al. 2019 Westermann et al. This content is distributed under the terms of the Creative Commons Attribution 4.0 International license.

**FIG 1:**
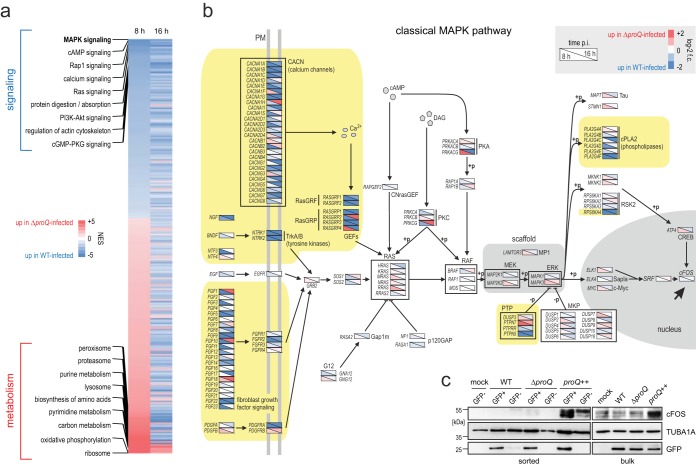
ProQ-dependent changes in *Salmonella*-infected HeLa cells. (a) Gene set enrichment analysis (GSEA) heat map showing human pathways that were significantly altered (FDR < 0.1; normalized enrichment score [NES] > 2 or < −2) between the infections of HeLa cells by wild-type (WT) or *proQ* deletion (Δ*proQ*) *Salmonella* strains. Some of the most drastically affected pathways are labeled. The results stem from two biological replicate experiments. (b) Schematic overview of the classical MAPK signaling pathway with comparative Dual RNA-seq data (Δ*proQ*- versus wild-type-infected HeLa cells; 8 and 16 h postinfection [p.i.]) plotted on top. The scheme was drawn manually using the human KEGG Pathview representation as the template. The yellow-shaded regions highlight branches of the pathway that were most severely affected (on the transcript level) by the absence of ProQ. Many of the downstream regulatory events are mediated by phosphorylation (+p), and thus, differences are not expected to be directly reflected in transcriptome data. Individual branches of the pathway converge at the activation of cFOS (black arrow). log-2 f.c., log_2_ fold change; PM, plasma membrane. (c) Western blot analysis confirms alterations in cFOS levels in differentially infected host cells. HeLa cells were infected (MOI of 50) with either wild-type *Salmonella* (WT), or the deletion (Δ*proQ*) or overexpression (*proQ*++) strain of ProQ—the two latter strains have highly similar invasion efficiencies and replication kinetics in this system (see Fig. S1d and e in the supplemental material) and are therefore directly comparable. Cells were harvested after 16 h p.i., sorted into invaded (GFP-positive) cells and uninfected (GFP-negative) bystanders or left unsorted (bulk), and total protein samples were separated on a 10% SDS-PAGE gel, transferred to a PVDF membrane, and probed with cFOS-specific antibodies. Tubulin (TUBA1A) serves as a human loading control, and detection of bacterial GFP confirms the purity of the sorted fractions.

Starting with the host transcriptome, *Salmonella* has been shown to evoke a proinflammatory response in epithelial cells (31, 32) which is thought to help this pathogen to access otherwise unavailable nutrients and outcompete other members of the gut microbiota (33, 34). A gene set enrichment analysis (GSEA) of differentially expressed host genes (Table S1A) revealed 266 human pathways that were significantly (FDR < 0.1) different upon infection with the Δ*proQ* mutant relative to wild-type infection (Fig. 1a). Generally, the lack of ProQ-mediated gene regulation in the infecting bacteria caused an upregulation of metabolic processes in the host, whereas host pathways involving immune, calcium, and G-protein signaling were downregulated, with mitogen-activated protein kinase (MAPK) signaling being the most strongly repressed host pathway.

Detailed inspection of human MAPK signaling component transcripts (Fig. 1b) pinpointed the most affected branches of the pathway and suggested that *Salmonella* ProQ is required to activate host cFOS, a constituent of the activator protein 1 transcription factor. On the protein level, we detected similar amounts of cFOS after infection with wild-type or Δ*proQ* mutant bacteria (Fig. 1c). However, overproduction of ProQ in the *Salmonella proQ*++ strain, which shows invasion efficiency and replication kinetics similar to those of the *Salmonella* Δ*proQ* strain in HeLa cells (Fig. S1d and e), led to a substantial increase in cFOS levels (Fig. 1c). Therefore, enhanced ProQ activity in infecting *Salmonella* increases MAPK signaling in infected epithelial host cells.

### ProQ effects in invading *Salmonella* involve motility, chemotaxis, and SPI-1 gene expression.

**FIG 2:**
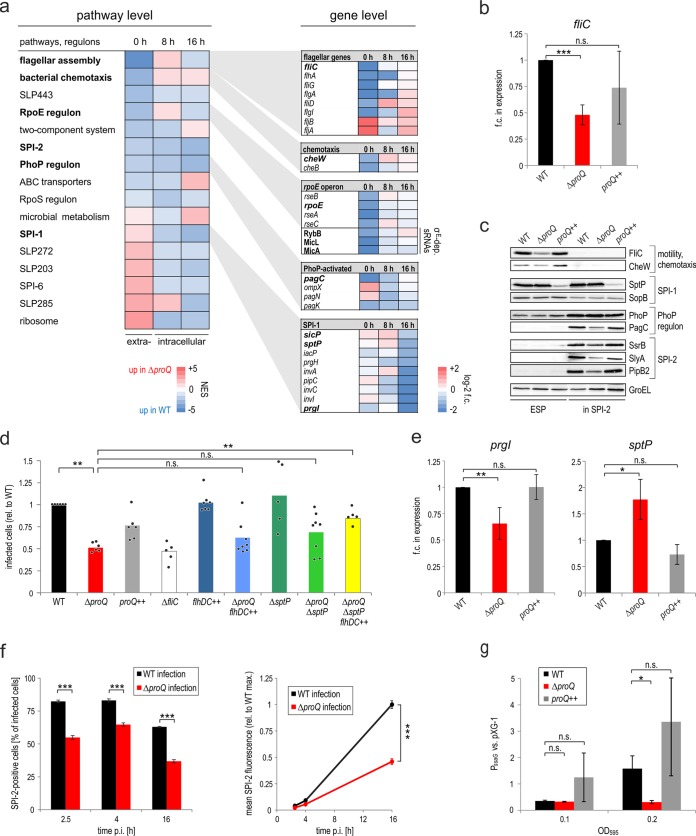
ProQ-dependent expression changes in infecting *Salmonella*. (a) Differential transcriptome analysis. (Left) GSEA pinpoints *Salmonella* pathways most severely affected by the absence of ProQ. Plotted are all pathways that were significantly altered (FDR < 0.1; normalized enrichment score [NES] > 2 or < −2) between wild-type and Δ*proQ Salmonella* for at least one time point. (Right) The significantly differentially expressed (FDR < 0.05) genes from several of the affected pathways are plotted. Genes with ProQ-dependent expression changes that were independently validated in this study are shown in bold type. The results stem from two biological replicate experiments. (b) Repression of *fliC* mRNA in the absence of ProQ. The indicated *Salmonella* strains were grown in LB to an OD_600_ of 2.0, total RNA was extracted and served as the template for qRT-PCR measurements using the constitutively expressed *gfp* mRNA as a reference. Values are means (bars) ± standard deviations (SD) (error bars) from four biological replicate experiments. (c) Western blot analyses demonstrate that ProQ-dependent differential virulence gene expression extends to the protein level. The indicated *Salmonella* strains were grown in LB to an OD_600_ of 2.0 (early stationary phase [ESP]) or in minimal SPI-2-inducing medium to an OD_600_ of 0.3 (in SPI-2), prior to the separation of total protein samples on a 10% SDS-PAGE gel. Membranes were probed with protein-specific (FliC, SopB, PagC, and GroEL) or FLAG-specific (other) antibodies. GroEL serves as loading control (one representative image is shown). (d) Influence of ProQ on *Salmonella* motility genes and on SptP expression is partially responsible for differential infection rates. HeLa infection rates of the indicated *Salmonella* strains. The *Salmonella* Δ*fliC* strain was included as a nonmotile control strain. The data are derived from flow cytometry measurements of infected (GFP-positive) cells relative to wild-type infection (individual replicate measurements shown as single dots; bars refer to the respective mean). Evaluation of significance was performed using a two-tailed nonparametric Mann-Whitney-Wilcoxon test. *P* values of ≤ 0.01 were considered very significant (**). n.s., not significant (*P* value > 0.05). (e) The mRNA of the T3SS-1 tip component PrgI is suppressed and that of the SPI-1 effector SptP is derepressed in the absence of ProQ. qRT-PCR measurements were performed preinfection (0 h; *prgI*) or at 1 h p.i. of HeLa cells (*sptP*; MOI of 50) on *proQ-*expressing versus non-*proQ*-expressing *Salmonella* strains. Constitutively expressed *gfp* mRNA served as a reference transcript. The data refer to the mean ± SD from five (*prgI*) or three (*sptP*) biological replicate experiments. (f) ProQ affects SPI-2 expression inside host cells. (Left) Cultured HeLa cells were infected (MOI 50) with *Salmonella* strains containing a transcriptional reporter of SPI-2 expression (P*_ssaG_*_::_*_gfp_*), in the respective *proQ* background. At the indicated time points, the fraction of SPI-2-positive relative to all infected HeLa cells was measured by flow cytometry. (Right) From the same experiment, the mean SPI-2 fluorescence level per SPI-2-positive host cell was quantified. Data refer to the mean ± SD from four independent experiments. (g) ProQ-dependent SPI-2 activation in defined minimal medium mimicking the vacuolar compartment. The indicated *Salmonella* strains were grown to an OD_595_ of 0.1 or 0.2, respectively, and the activity of the same transcriptional reporter as in panel f was measured and normalized against a constitutive GFP reporter (pXG-1). Data refer to the means ± SD from three independent experiments. In panels b, e, f, and g, significance was evaluated using a two-tailed Student’s *t* test and indicated as follows:*, *P < *0.05; **, *P < *0.01; ***, *P < *0.001; n.s., not significant.

**FIG 3:**
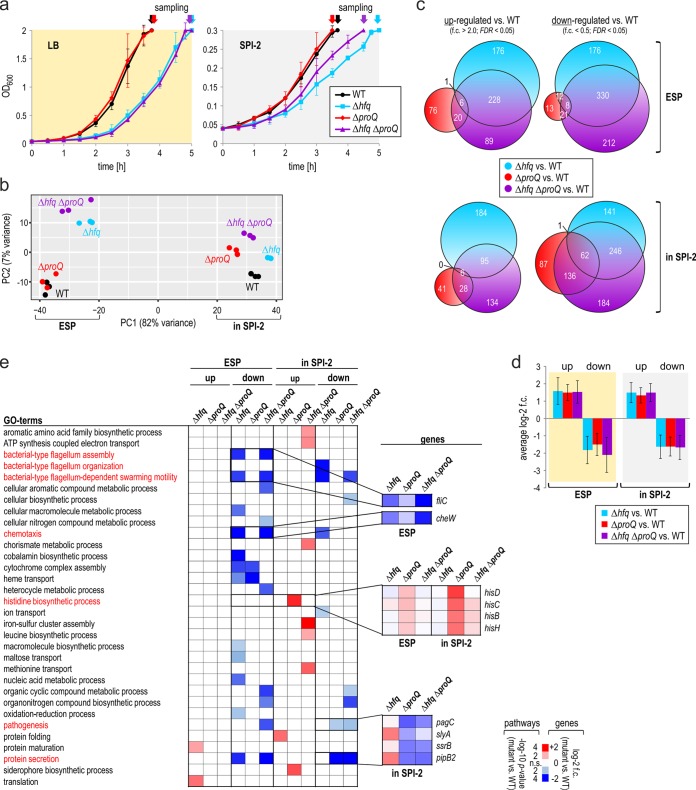
Comparison of the ProQ and Hfq regulons under infection-relevant conditions. (a) Growth curves of the four indicated *Salmonella* strains in LB or SPI-2-inducing medium. The graphs show the means ± SDs from three biological replicate experiments. The arrows indicate the sampling times of the cultures for total RNA extraction. (b) Principal-component analysis (PCA) plot of the RNA-seq data of the strains grown under the conditions shown in panel a (three replicates/condition). (c) Venn diagrams display the overlap of the ProQ and Hfq regulons. Plotted are all transcripts that were significantly differentially expressed (FDR < 0.05; log_2_ fold change [f.c.] > 1 or < −1) in the respective mutant compared to the wild-type background. (d) Average magnitude of the fold changes of the significantly differentially expressed genes (plotted in panel c) in the indicated mutants compared to the wild-type strain. (e, left) Gene Ontology (GO) term enrichment analysis of *Salmonella* pathways significantly affected (*P* value < 0.05; Bonferroni correction for multiple testing) in the respective RBP deletion backgrounds. (Right) Significantly differentially expressed (FDR < 0.05; log_2_ f.c. > 1 or < −1) genes from several of the affected pathways are plotted. The results stem from three biological replicate experiments.

Upon analyzing the impact of ProQ on *Salmonella* genes during host infection, we observed ∼200 mRNAs that were significantly (FDR < 0.05) differentially expressed between wild-type and Δ*proQ Salmonella,* at least at one of the three sampled time points (Table S1A). GSEA was used to identify the *Salmonella* pathways most severely affected by the absence of ProQ (Fig. 2a). At the time of invasion (0 h), the Δ*proQ* mutation caused downregulation of the genes encoding components of the motility and chemotaxis pathways as well as of the σ^E^ regulon and its associated sRNAs, whereas invasion (SPI-1) and ribosomal genes showed higher expression levels, despite a similar growth rate of the strains (Fig. 3a). Some of the most prominent ProQ-dependent expression changes during the invasion stage were independently validated. For example, the Dual RNA-seq results predicted a downregulation of the major flagellin FliC mRNA in the Δ*proQ* strain (Fig. S4a), and this was confirmed by qRT-PCR measurements (Fig. 2b) and Western blot analysis (Fig. 2c). Importantly, differential expression is likely due to a posttranscriptional effect of ProQ on *fliC* mRNA and not the result of genomic inversion of this phase-variable gene (Fig. S4b). The changes were dependent on ProQ since *proQ* complementation partially rescued *fliC* expression (Fig. 2b). Similar results were obtained for the transcripts of the chemotaxis gene *cheW* (Fig. S4c and d) and the *rpoE* operon (Fig. S4e, f, and g), including a slight regulation of CheW on the protein level (Fig. 2c). The reduced expression of motility and chemotaxis genes provided a possible explanation for the invasion defect observed for the Δ*proQ* mutant (Fig. S1d). However, attempts to restore flagellar gene expression through overexpression of the flagellar master regulator FlhDC (Δ*proQ*/*flhDC*++ strain) failed to restore wild-type invasion efficiency (Fig. 2d).

10.1128/mBio.02504-18.4 FIG S4 RNA-seq coverage plots and validation data for ProQ-dependent effects on *Salmonella* motility, chemotaxis, and the σ^E^ response. (a) Read coverage over the flagellar locus of *Salmonella* and zoom-in on the *fliC* gene. At the time of infection (0 h), *fliC* mRNA is downregulated in Δ*proQ Salmonella* (red) compared to wild-type *Salmonella* (black). (b) Orientation-specific PCR of the *fliC* locus of the indicated *Salmonella* strains using genomic DNA as the template. Information about the invertible region comes from reference 81. While all three strains contain populations of both *fliC*-ON and *fliC*-OFF bacteria, the majority express *fliC* (ON state). Importantly, the ratio (*fliC*-ON/*fliC*-OFF) is unchanged in the three strains. (c) Read coverage over the chemotaxis regulon and close-up view of the *cheW* locus. (d) qRT-PCR data for *cheW* mRNA. The indicated *Salmonella* strains were grown in LB to an OD_600_ of 2.0, total RNA was extracted and served as the template for qRT-PCR measurements. *gfp* mRNA was the reference transcript. Bars and error bars indicate the means ± SD from four biological replicate experiments. Significance was evaluated using a two-tailed Student’s *t* test (**, *P < *0.01; ***, *P < *0.001). (e) Read coverage over the *rpoE* operon and close-up view of the *rpoE* gene. (f) qRT-PCR data for *rpoE* mRNA. Data shown as described above for panel d. (g) Northern blot shows the expression of the σ^E^-dependent sRNAs RybB and MicA upon growth in LB to an OD_600_ of 2.0 (ESP) or in the minimal SPI-2-inducing medium to an OD_600_ of 0.3 (in SPI-2), respectively. Since both sRNAs were expressed at low levels in LB at an OD_600_ of 2.0, the respective membrane was reexposed for an extended time period (shown to the right). 5S rRNA serves as a loading control. Download FIG S4, PDF file, 0.3 MB. Copyright © 2019 Westermann et al. 2019 Westermann et al. This content is distributed under the terms of the Creative Commons Attribution 4.0 International license.

The absence of ProQ also had divergent effects on individual SPI-1-associated transcripts. For example, the *prg* operon encoding the T3SS-1 was downregulated in invading Δ*proQ* versus wild-type *Salmonella* (i.e., the 0-h time point), whereas the *sicP*-*sptP* transcriptional unit encoding the SPI-1 effector SptP and its chaperone SicP were expressed at higher levels than in wild-type *Salmonella* (Fig. 2a; Fig. S5a). These Dual RNA-seq-based predictions for the *prgI, sicP*, and *sptP* mRNAs were independently confirmed by qRT-PCR (Fig. 2e; Fig. S5b). Notably, the mRNA for the major SPI-1 transcriptional activator HilD was largely unaffected by ProQ status (Fig. S5b), indicating that ProQ acts directly on the *prgI* and *sicP*-*sptP* mRNAs. This is further supported by our previous observation that the *prgHIJK* and *sicP*-*sptP* mRNAs, but not the *hilD* mRNA, can be cross-linked to ProQ (22) (Table S1B).

10.1128/mBio.02504-18.5 FIG S5 RNA-seq coverage plots and validation data for ProQ-dependent expression changes of *Salmonella* pathogenicity islands. (a) Read coverage over the SPI-1 locus of *Salmonella* and zoom-in onto the TSS of the *sicP*-*sptP* transcript. TSS annotation (black arrow) obtained from reference 64. (b) qRT-PCR data on the expression of selected *Salmonella* SPI-1 virulence genes (the *sicP*-*sptP* operon and the mRNA of the SPI-1 master regulator, *hilD*) in *proQ*-expressing versus non-*proQ*-expressing strains during the infection of HeLa cells (MOI of 50). *gfp* mRNA was used as a reference. The plot displays the mean ± SD from three biological replicate experiments. (c) Read coverage over the SPI-2 locus. (d) qRT-PCR data on the expression of selected SPI-2 genes (a gene for a secreted effector, *pipB2*; the gene for the SPI-2 master regulator, *ssrB*) in *proQ*-expressing versus non-*proQ*-expressing strains during the infection of HeLa cells (MOI of 50). *gfp* mRNA was the reference. The data are the means ± SD from three biological replicate experiments. Download FIG S5, PDF file, 1.4 MB. Copyright © 2019 Westermann et al. 2019 Westermann et al. This content is distributed under the terms of the Creative Commons Attribution 4.0 International license.

Upon their translocation, certain SPI-1 effectors (namely, SopE, SopE2, and SopB) cooperate to reorganize the host actin cytoskeleton, enabling *Salmonella* to invade epithelial cells (35–37). Translocated SptP protein subsequently counteracts the activities of these effectors, helping to reorganize the cytoskeleton to preinfection conditions (36, 38). This suggested that elevated SptP secretion could, at least in part, be the basis for the invasion defect of the Δ*proQ* strain (Fig. S1d). To test this, we first deleted the *sptP* gene alone (Δ*proQ*/Δ*sptP* strain) yet failed to see significant improvement of the invasion efficiency (Fig. 2d). However, combining *sptP* deletion with flagellar overexpression (Δ*proQ*/Δ*sptP*/*flhDC*++ strain) did significantly increase the invasion rate of the Δ*proQ* strain, albeit not to the level of wild-type *Salmonella* (Fig. 2d). Therefore, the impaired invasion rate of the *Salmonella* Δ*proQ* strain is a multifactorial phenotype and at least partially due to the reduced expression of motility genes and enhanced levels of the SptP effector.

### ProQ affects SPI-2 expression and the σ^E^ response in intracellular *Salmonella*.

To understand the role of ProQ after successful host cell invasion, we analyzed the two intracellular stages (8 h and 16 h p.i.) of the Dual RNA-seq data (Table S1A). Host cell invasion is generally accompanied by widespread expression changes in the infecting *Salmonella*, including the upregulation of metal ion uptake systems and induction of the envelope stress response (30, 39–41). Interestingly, envelope stress was differentially affected by the absence of ProQ with lower steady-state levels of σ^E^-dependent sRNAs, RybB and MicA, in Δ*proQ Salmonella* (Fig. S4g). Additionally, the SPI-2 locus was exclusively activated once *Salmonella* reached its intracellular niche (Fig. S5c). Interestingly, SPI-2 expression was slightly reduced in the Δ*proQ* strain compared to the wild-type strain (Fig. 2a; Fig. S5c). qRT-PCR measurements during the infection of HeLa cells revealed only marginal differences in the expression of selected SPI-2 genes (the gene for the secreted effector PipB2 and that for the SPI-2 master regulator SsrB) in the absence of ProQ. However, overproduction of ProQ substantially enhanced intracellular SPI-2 activation. Likewise, a ProQ-dependent effect on PipB2 protein levels was observed *in vitro* by Western blot analysis (Fig. 2c).

To further support the positive effect of ProQ on SPI-2 activity, cultured HeLa cells were infected with *Salmonella* strains containing a robust transcriptional reporter of SPI-2 expression (*P_ssaG_*::*gfp*) in the respective *proQ* background (Fig. 2f). In this reporter, GFP expression is driven by the promoter of the SsrB-activated gene *ssaG* (42), and thus, GFP intensity serves as a proxy for SPI-2 activity. At all three time points measured (2.5, 4, and 16 h p.i.), the fraction of SPI-2-positive HeLa cells was lower for Δ*proQ* bacterial infections than for wild-type infections (Fig. 2f, left). Likewise, mean GFP intensity (corresponding to mean SPI-2 activity) per SPI-2-positive host cell was reduced in the absence of ProQ (Fig. 2f, right). A similar attenuation in SPI-2 induction in the Δ*proQ* background as well as an (over)complementation in the *proQ*++ background were observed in an infection-relevant *in vitro* assay. Here, *Salmonella* bacteria were grown to defined densities in minimal SPI-2-inducing medium (42), and *ssaG* promoter activity was measured as a proxy for SPI-2 induction (Fig. 2g).

In summary, ProQ affects the expression of *Salmonella* motility and virulence genes during infection. Of note, *fliC* and *cheW* mRNAs as well as selected SPI-1 and SPI-2 transcripts have previously been identified as direct ProQ ligands in cross-linking experiments (22) (Table S1B), arguing for a direct effect of ProQ binding on the steady-state levels of these transcripts.

### Comparative analysis of the ProQ and Hfq regulons under infection-relevant conditions.

To understand the extent of the ProQ-dependent regulations, we compared the identified genes with the regulon of the other major RNA chaperone of *Salmonella*, Hfq. Importantly, several of the flagellar and virulence genes affected by ProQ have previously been shown to be dysregulated in a *Salmonella* Δ*hfq* strain (9, 10). To avoid secondary effects caused by the different invasion efficiencies of Δ*proQ* and Δ*hfq* bacteria, we performed RNA-seq analysis of bacteria grown in medium that mimics invasion (early stationary phase [ESP] in LB) or intracellular replication (defined minimal SPI-2-inducing medium [42]). Under these conditions, the *proQ* deletion mutant grew indistinguishably from wild-type *Salmonella*, whereas the Δ*hfq* strain displayed a considerably longer lag phase (in LB medium) or slightly reduced growth (SPI-2-inducing conditions) (Fig. 3a).

Principal-component analysis of the RNA-seq data (Fig. 3b) revealed two major clusters, splitting the samples along principal component 1 (PC1), reflecting the two growth conditions (ESP versus “in SPI-2”). Additionally, within the ESP cluster, the Δ*proQ* bacteria colocalized with the wild-type *Salmonella* but separated from Δ*hfq* and the Δ*proQ* Δ*hfq* double mutant. In contrast, in SPI-2-inducing medium, the wild-type and Δ*proQ* transcriptomes were segregated from each other, suggesting that ProQ may exert stronger effects on the *Salmonella* transcriptome in intracellular bacteria compared to invading bacteria. Likewise, the absolute numbers of significantly (FDR < 0.05) differentially expressed genes in Δ*proQ* compared to wild-type *Salmonella* were higher in SPI-2 than in ESP, albeit the total number of differentially expressed genes was lower than the number of Hfq-dependent regulated genes (Fig. 3c; Table S1C). Notwithstanding this, the average magnitude of the fold changes of differentially expressed genes in the respective mutant backgrounds was highly similar between the Hfq and ProQ regulon (Fig. 3d). Of note, the set of genes differentially expressed in the Δ*proQ* Δ*hfq* double mutant was not simply the sum of genes deregulated in the two single mutant strains (Fig. 3c), suggesting some degree of synergy between these two major RBPs.

Pathway analysis of the differentially expressed genes (Fig. 3e) recapitulated many of the findings from the comparative Dual RNA-seq screen for ProQ (Fig. 2a). Interestingly, however, motility and chemotaxis pathways were even more strongly repressed in the absence of Hfq compared to ProQ, and again more so in the absence of both chaperones, suggesting additive effects of Hfq and ProQ on the respective transcript abundance. In contrast, SPI-2 genes were specifically repressed in the absence of ProQ (Δ*proQ* and Δ*proQ* Δ*hfq* strains) but not affected by the deletion of *hfq*. Another ProQ-specific footprint was revealed with respect to genes involved in histidine biosynthesis. Interestingly, these genes were derepressed in the absence of ProQ which was partially rescued by removing Hfq, suggesting that the two major chaperones might fulfill opposing functions in the regulation of histidine biosynthesis (note, however, that SL1344, the *Salmonella* strain used in this study, is a histidine auxotroph [43]).

### Screen for infection-relevant ProQ-dependent sRNAs.

**FIG 4:**
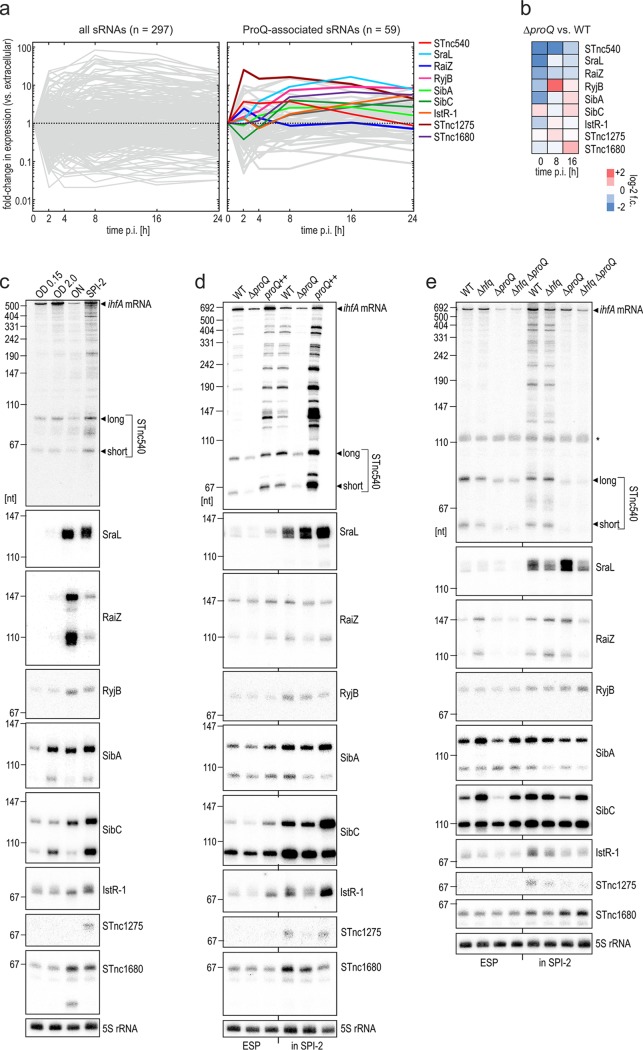
Screen for infection-induced ProQ-dependent sRNAs. (a) Dual RNA-seq data on the expression of ProQ-associated sRNAs (as defined in references 13 and 22) during a high-resolution time course of the infection of HeLa cells by wild-type *Salmonella*. Expression data were retrieved from a previous study (30) (accession number GSE117256). (b) Comparative expression of ProQ-associated infection-induced sRNAs (colored in panel a) between Δ*proQ* strain and the wild-type *Salmonella* at the indicated time points after HeLa infection. The results stem from two biological replicate experiments. (c) Northern blot on the expression of the denoted sRNAs. Total RNA samples were taken from wild-type *Salmonella* grown under the indicated *in vitro* conditions, separated on a 6% PAA/urea gel, blotted, and probed with sRNA-specific DNA oligonucleotides (Table S1G). OD, optical density (in LB); ON, overnight growth in LB; SPI-2, SPI-2-inducing conditions (see Materials and Methods). (d and e) Northern blot data for the same sRNAs in the presence or absence of ProQ (d) or Hfq (e) in ESP (LB; OD 2.0) or SPI-2-inducing conditions. Detection was performed as described above for panel c. 5S rRNA serves as loading control in panels c to e.

ProQ not only binds hundreds of different mRNAs but also binds close to 50 sRNAs, most of which are of uncharacterized function. To obtain a better understanding of the expression of these ProQ-associated sRNAs (13, 22), we reanalyzed our previous high-resolution Dual RNA-seq data of intracellular *Salmonella* (30). Figure 4a summarizes the expression kinetics of these ProQ-associated sRNAs during a 24-h time course of HeLa cell infection. Interestingly, the maximal fold changes in their expression (Fig. 4a, right) are about an order of magnitude lower than for ProQ-independent sRNAs (Fig. 4a, left). We identified three sRNAs—SraL (44, 45), RaiZ (23), and the uncharacterized 3′-derived sRNA STnc540 (7)—for which expression was consistently reduced in ProQ-deficient *Salmonella* grown in HeLa cells (Fig. 4b; Table S1A).

The expression of SraL, RaiZ, and STnc540 as well as six additional intracellularly induced (30) ProQ-associated sRNAs in wild-type *Salmonella* during various growth stages *in vitro* (Fig. 4c) was analyzed by Northern blotting. STnc540 levels, for which we detected two main species (∼90 and ∼60 nt), did not necessarily correlate with the expression of its parental transcript, the *ihfA* mRNA encoding integration host factor α (Fig. 4c). For example, STnc540 expression peaked under SPI-2-inducing conditions, while maximal *ihfA* expression occurred in early exponential phase (OD of 2.0), suggesting that different factors determine the steady-state levels of the sRNA and mRNA. Confirming previous results (23), however, the 3′-derived sRNA RaiZ accumulated in stationary-growth bacteria as both a long (∼160-nt) and short (∼120-nt) isoform (Fig. 4c). The intergenic sRNA SraL accumulated in both stationary phase and in SPI-2 medium (Fig. 4c), which agrees with SraL being a σ^S^-dependent sRNA (44) and its ∼25-fold induction during intracellular replication (Fig. 4a). Similarly, the antisense-encoded RyjB, the sense-overlapping STnc1680, as well as the antitoxin RNAs IstR-1, SibA, and SibC were induced in stationary phase and under SPI-2 conditions, whereas the candidate sRNA STnc1275 was exclusively detected in SPI-2 medium (Fig. 4c).

We expanded this Northern blot analysis by including the Δ*proQ* and *proQ*++ strains (Fig. 4d). This confirmed that ProQ is generally required for full expression of STnc540 and RaiZ, whereas its effect on SraL was limited to growth in LB medium (ESP) (Fig. 4d). This agrees with the RNA-seq data, which suggested an effect of ProQ on SraL levels primarily prior to infection (Fig. 4b). Of the other sRNAs, expression of IstR-1, STnc1275, and SibC positively correlated with ProQ. In contrast, RyjB and SibA showed unchanged steady-state levels in the absence of ProQ, despite them being *bona fide* ligands of this RBP (22). Finally, STnc1680 levels were negatively affected by both the absence of ProQ and its overexpression. This highlights that for many of the tested sRNAs, the function of ProQ is likely more complex than mediating sRNA stabilization.

Finally, we investigated the potential role of Hfq on the expression of the above-described sRNAs. While Hfq slightly affected the abundance of most of the sRNAs, STnc540 was exclusively affected by ProQ (Δ*proQ* and Δ*proQ* Δ*hfq* strains; Fig. 4e). As this suggested STnc540 to be a fully ProQ-dependent and Hfq-independent sRNA, we selected it for further characterization.

### Interplay between STnc540 and ProQ affects expression of a magnesium importer.

**FIG 5:**
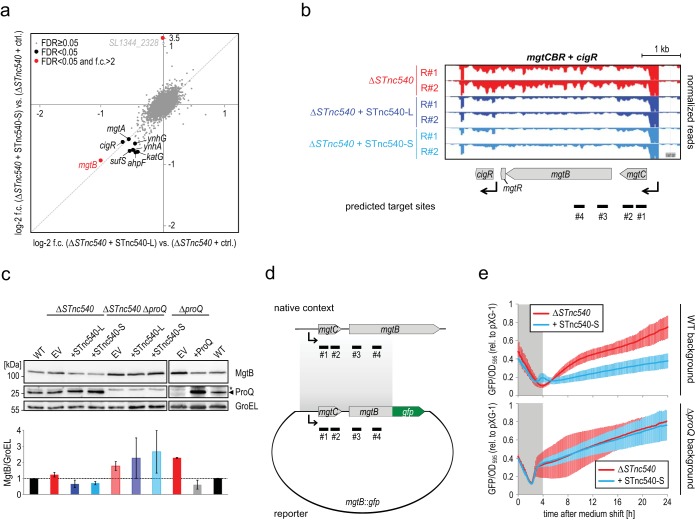
STnc540 target identification screen. (a) Pulse expression of STnc540 under SPI-2 conditions. Either one of the two STnc540 isoforms was overexpressed for 10 min prior to total RNA isolation and sequencing. The log_2_ fold changes in expression upon induction of the long (*x* axis) or short (*y* axis) STnc540 isoform compared to an isogenic strain carrying the empty control vector are plotted. The results represent the means from two biological replicate experiments. mRNAs whose expression was significantly altered (FDR < 0.05) upon induction of at least one STnc540 isoform compared to the control are labeled. *SL1344_2328* is a low-confidence target (as inferred from manual read coverage inspection; not shown). (b) Read coverage plot of the expression of the *mgt* locus upon pulse expression of the long (dark blue) or short (light blue) STnc540 isoform relative to the empty control vector. The position of predicted interaction sites with STnc540 (Fig. S6d) are denoted. TSS annotations (black arrows) were derived from reference 64. (c) Western blot analysis indicates a slight repression of MgtB protein levels upon constitutive overexpression of STnc540 in the presence of ProQ. Wild-type, Δ*STnc540*, Δ*proQ*, or Δ*STnc540* Δ*proQ Salmonella* with 3xFLAG-tagged MgtB expressed from its native promoter and harboring the indicated, constitutive sRNA overexpression plasmids or pZE-ProQ, respectively, were grown under SPI-2-inducing conditions to an OD_600_ of 0.5. Total protein samples were harvested and analyzed by Western blotting using FLAG-specific or endogenous ProQ-detecting antibodies (the position of ProQ is indicated by an arrowhead; the asterisk denotes an unspecific signal). GroEL serves as a loading control. A representative Western blot experiment out of three independent biological replicates is shown, and the quantification of the MgtB signal intensity in the different strains (normalized to GroEL and relative to the wild-type strain) over the three replicates is given in the graph below the Western blot. (d) Schematic representation of the *mgtB*::*gfp* reporter construct used in panel f. The predicted interaction sites with STnc540 (#1 to #4; see Fig. S6d) are indicated. (e) GFP reporter assay for STnc540-dependent regulation of *mgtB*. The reporter construct in panel d was cotransformed with a constitutive STnc540 (short isoform) overexpression plasmid or the respective vector control into a Δ*STnc540* (top) or Δ*STnc540* Δ*proQ* (bottom) *Salmonella* background. The resulting strains were grown under SPI-2-inducing conditions in a 96-well plate for 24 h, and the GFP intensity (as a proxy for MgtB levels) was monitored in 10-min intervals. The data show the means ± SD of the GFP intensity normalized to OD_595_ values and relative to the GFP intensity of the same strains harboring a constitutive GFP expression control plasmid (pXG-1) from three biological replicates, each comprising technical triplicates. In the first 3-4 h, OD, but not GFP intensity, increased (grey windows).

Given that ProQ is an RNA chaperone and that previously characterized ProQ-associated sRNAs all function by base pairing mechanisms with other cellular RNAs (23, 44), we opted for an sRNA pulse expression approach (46, 47) to identify potential STnc540 RNA targets. To perform this in a STnc540 null background, we removed most of the STnc540 sequence from the *Salmonella* chromosome while maintaining the terminator for *ihfA* mRNA (Δ*STnc540* strain; Fig. S6a and b). The resulting mutant strain did not show any significant growth difference compared to the parental wild-type strain (Fig. S7). STnc540 was subsequently induced from a plasmid-borne promoter under the condition of maximal expression of the endogenous sRNA, i.e., under SPI-2-inducing conditions (Fig. 4c). RNA samples from the sRNA overexpression strains (the long or short STnc540 version) were collected 10 min after induction, analyzed by RNA-seq, and compared to samples from a control strain in which the empty vector was induced (Fig. 5a; Table S1D). Of the 2,312 mRNAs captured (≥100 aligned reads/gene), eight were downregulated >1.5-fold upon overexpression of STnc540. There was a high correlation between the RNA-seq data for the long and short STnc540 isoforms, arguing that the short sRNA version possesses the full regulatory capacity.

10.1128/mBio.02504-18.6 FIG S6 Construction of an STnc540 deletion mutant and *in silico* prediction of target interactions. (a) To generate the Δ*STnc540* strain, the 5′ portion of the sRNA gene was removed, while its terminator was kept. TSS annotations (black arrows) are according to reference 64. (b) Northern blot validation of the successful deletion of STnc540. *Salmonella* strains were grown in LB to stationary phase (ON) or under the SPI-2-inducing condition, and total RNA samples were analyzed by Northern blotting using probes against the 3′ end of STnc540 or the body of the *ihfA* mRNA. 5S rRNA serves as a loading control. (c) qRT-PCR for *mgtB* mRNA at the indicated time points upon the addition of arabinose to strains harboring the long or short STnc540 isoform on the pBAD plasmid relative to the control strain harboring the empty vector. *gfp* mRNA was the reference. The data are means ± SD from three biological replicate experiments. Significance (*, *P* value of <0.05) was evaluated using a two-tailed Student’s *t* test. (d) *In silico* prediction of RNA-RNA interactions between STnc540 and its putative target *mgtCBR*. *RNAhybrid* (49) was used to predict interaction sites. The numbering of nucleotides is relative to the respective TSS of the mRNA or the annotated 5′ end of STnc540, respectively, based on the annotations from reference 64. Download FIG S6, PDF file, 1.4 MB. Copyright © 2019 Westermann et al. 2019 Westermann et al. This content is distributed under the terms of the Creative Commons Attribution 4.0 International license.

10.1128/mBio.02504-18.7 FIG S7 Growth of different mutant strains of *Salmonella*. The indicated strains were grown in LB (left) or minimal SPI-2-inducing medium (right), and OD_600_ values were measured in 30-min intervals. The data are means ± SD from two biological replicate experiments. Download FIG S7, PDF file, 1.3 MB. Copyright © 2019 Westermann et al. 2019 Westermann et al. This content is distributed under the terms of the Creative Commons Attribution 4.0 International license.

The top candidate target was the *mgtB* mRNA encoding a magnesium import protein (Fig. 5a and b). The gene is part of the *mgtCBR* locus that is silent in rich medium but whose transcription is activated by the two-component system PhoP/Q under SPI-2 conditions (48). Interestingly, the downregulation upon STnc540 induction selectively applied to *mgtB* and not to the other members of this operon. Using the *RNAhybrid* software (49), we predicted possible RNA-RNA interactions between STnc540 and *mgtB* (Fig. S6d). Notably, in three of the four proposed interactions (#2 to #4 in Fig. S6d), the 3′ region of STnc540 where ProQ binds (22), was engaged in base pairing. This may explain why the short sRNA isoform is sufficient for target repression.

In the pulse expression experiment above, either isoform of STnc540 reduced the level of the *mgtB* mRNA to ∼50% (Fig. 5a; Fig. S6c). On the level of the MgtB protein which we rendered detectable via addition of a chromosomal 3xFLAG tag, repression by constitutively expressed STnc540 was slightly less pronounced (Fig. 5c). Importantly, however, removing both STnc540 and ProQ substantially increased the basal levels of MgtB-3xFLAG (compared to the wild type), and abrogated the reduction of MgtB when STnc540 was coexpressed in the Δ*proQ* background (Fig. 5c). Furthermore, STnc540 repressed a fluorescent reporter of MgtB, harboring the 5′ portion of its open reading frame fused to GFP (Fig. 5d and e; Fig. S8a). In contrast, removing the putative interaction sites #3 and #4 from this construct (*mgtC*::*gfp* in Fig. S8a) abrogated repression by STnc540, arguing that one or both of these sites may be the actual base-pairing region(s) with STnc540.

10.1128/mBio.02504-18.8 FIG S8 GFP reporter assays for STnc540/ProQ-mediated control of MgtB. Schematic representation (top) and measurement (bottom) of a transcriptional reporter (P*_mgt_*::*gfp*) and two transcriptional/posttranscriptional reporters (*mgtC*::*gfp* and *mgtB*::*gfp*) for which the open reading frame of GFP was fused to the *mgt* locus at the indicated positions. The predicted interaction sites with STnc540 (#1 to #4; see Fig. S6d) are indicated below the schematic representations. These reporter constructs were cotransformed with a constitutive STnc540 (long or short isoform) overexpression plasmid, the ProQ expression plasmid, or the respective vector control into the Δ*STnc540* (a) or Δ*STnc540* Δ*proQ* (b) *Salmonella* background. The resulting strains were grown in SPI-2-inducing medium in a 96-well plate for 24 h, and the GFP intensity (as a proxy for MgtC or MgtB levels) was monitored in 10-min intervals. The data show the means ± SD of the GFP intensity normalized against OD_595_ and relative to the GFP intensity of the same strains harboring a constitutive GFP expression control plasmid (pXG-1) from three biological replicates, each comprising technical triplicates. Download FIG S8, PDF file, 1.6 MB. Copyright © 2019 Westermann et al. 2019 Westermann et al. This content is distributed under the terms of the Creative Commons Attribution 4.0 International license.

Most importantly, the repression of the *mgtB*::*gfp* reporter was abrogated in the Δ*proQ* background (Fig. 5d and e; Fig. S8b), supporting the basic requirement of ProQ for STnc540-mediated repression of the *mgtB* mRNA. Interestingly, ProQ delayed the induction of the *mgtB*::*gfp* reporter in the absence of STnc540 (Fig. S8b), arguing that this RBP, directly or indirectly, counteracts *mgtB* expression by an additional, STnc540-independent, mechanism. Although more mechanistic analysis is needed to dissect the regulatory interplay between STnc540, ProQ, and *mgtB*, our results support the idea that ProQ engages directly in the regulation of *Salmonella* virulence genes.

## DISCUSSION

With ProQ biology being in its infancy (19), a fundamental question remains as to whether and how the global RNA-binding activity of this RBP, which in *Salmonella* includes many interactions with transcripts from major virulence regions (22), impacts pathogenesis. The present study reveals that ProQ is required for optimal virulence gene expression in *Salmonella* and directly compares the molecular consequences of loss of function of ProQ with those of the primary enterobacterial sRNA-binding chaperone, Hfq.

### Molecular consequences of the loss of ProQ for *Salmonella* pathogenesis.

In *Salmonella*, ProQ controls motility and chemotaxis gene expression and affects specific SPI-1 transcripts. ProQ further exerts a positive influence on the expression of members of the PhoP regulon and, particularly, SPI-2 genes, implicating ProQ in the cross talk between the two major pathogenicity islands of this bacterium, SPI-1 and SPI-2. Overall, the impact of ProQ on *Salmonella* expression of coding genes is most likely a combination of the dysregulated expression of direct mRNA ligands (such as *cspE* mRNA [22]) and indirect derepression of mRNAs normally targeted by ProQ-associated sRNAs (such as *hupA* mRNA [23]). It is important to note that ProQ binding can have divergent outcomes for different RNA ligands, even if they belong to the same regulon. For example, ProQ targets transcripts of both the SPI-1-controlled operons *prgHIJK* and *sicP*-*sptP* (22). However, we found here that the deletion of *proQ* reduced the steady-state levels of *prgI* mRNA, while it enhanced *sptP* expression. Therefore, more insight into the potentially diverse molecular mechanisms of ProQ is needed to fully correlate ProQ binding with changes in gene expression.

**FIG 6:**
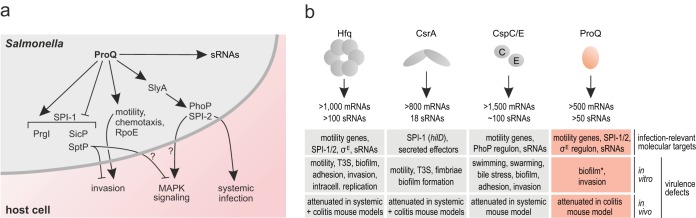
Roles of global RBPs in *Salmonella* virulence. (a) Model of infection-relevant regulations mediated by ProQ that were uncovered in the present study. (b) Known contributions of the five global RBPs for *Salmonella* virulence mechanisms and phenotypes associated with their respective deletion. Information about the number of RNA ligands for the indicated proteins is derived from references 5, 6, 13, and 22. E. coli mutants devoid of *proQ* exhibit defects in biofilm formation (70) (indicated by biofilm*). See main text for further information.

On the side of the infected host, this transcriptome analysis revealed that the Δ*proQ* mutant elicits less MAPK signaling compared to wild-type *Salmonella.* Notably, the MAPK signaling cascade, which activates nuclear factor κB (NF-κB) and interferon responses, is one of the prime targets of bacterial pathogens as they manipulate host immunity for their own benefit (50), and several *Salmonella* effector proteins are known to target MAPKs (51). For example, specific effectors of the SPI-2 regulon—expression of which is reduced in *Salmonella* Δ*proQ* bacteria—are known activators of MAPK signaling in the host (52–54), whereas the SPI-1 effector SptP, whose level increases in the absence of ProQ, inhibits the MAPK cascade through ERK (extracellular signal-regulated kinase) (55). Therefore, the ProQ-dependent expression changes in the bacterial transcriptome provide an explanation for the observed differences between the host response to wild-type and Δ*proQ Salmonella* (Fig. 6a).

### Comparing the ProQ and Hfq regulons under virulence conditions.

Hfq and ProQ are the two major sRNA-binding proteins of *Salmonella* (13), and each of them targets hundreds of different transcripts with little overlap, at least when assayed in early stationary-phase cells (5, 22). Strikingly, however, our comparative transcriptomics here revealed an unexpected overlap between the ProQ and Hfq regulons under infection-relevant conditions. Key virulence systems, such as the flagellum or the SPI-1 secretion system, were dysregulated in the absence of either one of these RNA chaperones. However, interactome studies suggest that different regulatory cascades are affected by ProQ and Hfq and that these branches converge at the level of flagellin expression or SPI-1 secretion, respectively. For example, Hfq binds *flhDC*, encoding the master regulator of flagellar expression (5), while ProQ directly interacts with the major flagellin-encoding *fliC* mRNA (22). Likewise, Hfq (5), but not ProQ (22), binds the mRNA for the SPI-1 master regulator HilD. Instead, ProQ directly binds to individual SPI-1 transcripts for structural components of the SPI-1 needle or secreted effectors (e.g., the *prg* and *sicP*-*sptP* operons) (22). In summary, despite overall similar virulence processes being affected by both global RBPs, the higher-resolution dissection on transcript level reveals that Hfq and ProQ target distinct branches in the corresponding pathways.

Importantly, the positive effect on SPI-2 genes was exclusive to ProQ, whereas the deletion of *hfq* had no effect on SPI-2 expression. As ProQ interacts with the mRNAs of two key regulators of the SPI-2 pathway, SlyA and SsrB (22), we probed the levels of these proteins in the presence or absence of the RBP. While SsrB levels were minimally affected, SlyA abundance decreased in Δ*proQ Salmonella*, proposing the latter as the relevant target through which ProQ promotes SPI-2 expression. We note, however, that former RIP-seq (13) and CLIP-seq data (22) were obtained from *Salmonella* grown to exponential phase in LB, i.e., a condition under which SPI-2 genes are poorly expressed. Therefore, our comparative expression data should motivate future ProQ interactome studies under similar *in vitro* conditions which better reflect the intracellular lifestyle of this pathogen.

### Identification of infection-relevant ProQ-dependent sRNAs.

Our Dual RNA-seq experiments revealed a set of infection-induced sRNAs whose levels depend on ProQ (Fig. 4a and b) and which include STnc540. This sRNA is made from the 3′ UTR of the *ihfA* mRNA which encodes the α-subunit of the conserved, heterodimeric integration host factor (IHF) complex. The protein made from the mRNA is important for *Salmonella* pathogenesis: by alleviating H-NS-mediated transcriptional repression, IHF contributes to the activation of virulence genes (56, 57). Interestingly, the experimental target screen for STnc540 suggests that the noncoding region of the *ihfA* mRNA may also be involved in the regulation of virulence genes. At least when overexpressed, the STnc540 sRNA selectively represses the mRNA of the MgtB magnesium ion transporter.

Regulation of *mgt* genes could hardly be more complex. Early work showed that *mgtCBR* expression is transcriptionally activated via PhoQ/P (48). However, subsequent work (58–63) revealed multiple additional regulatory layers that make expression of the *mgt* operon responsive to disparate external and internal cues. Among those, a PhoP-activated *cis*-antisense RNA (AmgR) constitutes a negative-feedback loop to counteract *mgtCBR* expression in an Hfq- and RNase E-dependent manner. Additionally, two short open reading frames (termed *mgtM* and *mgtP*) within the long *mgtC* leader respond to increasing ATP or decreasing proline levels, respectively, and control translation elongation into *mgtCBR* via an attenuation-like mechanism.

Here, we identified STnc540 as the first *trans*-acting riboregulator of *mgtB*. We hypothesize that STnc540 may serve as a molecular device setting an upper limit to the expression of this infection-induced transporter. It should be noted, however, that *mgtB* mRNA was not identified in previous *in vivo* mappings of ProQ ligands, the caveat being that *mgtB* was hardly expressed under those conditions (13, 22, 64). Alternatively, ProQ may interact only with STnc540 to stabilize and/or unwind the sRNA, that is, without making contact with the target mRNA. This would be different from Hfq-dependent regulations which usually involve the formation of Hfq-sRNA-mRNA complexes. However, it would also differ from the previously reported ProQ-dependent regulation of *hupA* mRNA by the RaiZ sRNA in which a trimeric complex with ProQ is formed (23). Similarly, the ProQ-related RocC protein in Legionella pneumophila is also assumed to bind both the RocR sRNA and its mRNA targets (24).

It also remains to be fully established whether *mgtB* is a physiologically relevant target of STnc540. Admittedly, the observed ∼2-fold decrease in *mgtB* mRNA and MgtB protein levels upon ectopic STnc540 expression is moderate. However, the STnc540/*mgtB* pair has value because it increases the currently small number of target transcripts for the study of the molecular mechanisms of ProQ-mediated regulation. Importantly, our preliminary results suggest that STnc540 binds *mgtB* mRNA in the coding sequence where sRNAs have to compete with elongating ribosomes and for which few cases of sRNA-mediated regulation are currently known (65). This mechanism would also be different from the ProQ/RocC-mediated regulations by the RaiZ/RocR sRNAs which similarly to Hfq-associated sRNAs sequester the ribosome binding sites of their target mRNAs (23, 24). In addition to the intergenic and UTR-derived sRNAs that were inspected in the present study, recent observations imply that stable RNAs may also be carved out from coding regions (87) and some of these “decay-generated noncoding RNAs” in *Salmonella* bind ProQ. What relevance these RNAs might have for physiology and virulence remains an open question for future efforts.

### What is the role of ProQ *in vivo*?

The observed infection defects notwithstanding, the general robustness of *Salmonella* virulence in the face of the lack of the central RNA modulator ProQ is astonishing. A concept referred to as “phase transition” was recently proposed for eukaryotic RBPs (66). According to this hypothesis, surplus cellular transcripts may localize in distinct speck-like complexes together with RBPs, and thus be sequestered in a nonfunctional state. Upon detection of a specific stimulus, these specks can rapidly dissolve, giving rise to an immediate pool of ready-to-use transcripts. If bacterial RBPs may work in a similar fashion, one would expect rather mild phenotypic effects upon their deletion, as reported here for Δ*proQ Salmonella*. The major consequence of ProQ removal in this case would be that more or less RNA is stored in the corresponding complexes, whereas the steady-state levels of free (i.e., functional) RNA would hardly be affected. Future efforts, e.g., subcellular localization studies, should address whether ProQ might fulfill such an RNA-buffering function during *Salmonella* infection.

Alternatively, simplistic cell culture models reflect host complexity only to a certain degree. It is not unheard of that bacterial mutant strains with a weak macroscopic virulence defect *in vitro* still produce a robust phenotype in whole-animal models (67). In a first attempt to assess the impact of ProQ on *Salmonella* virulence in an animal model, we compared the colonization ability of the Δ*proQ* strain to that of wild-type bacteria after oral infection of streptomycin-pretreated C57BL/6 mice (see Fig. S9 in the supplemental material). This revealed an attenuation in survival/growth of ProQ-deficient *Salmonella* at systemic sites, although not reaching the level of a functional SPI-2 knockout (Δ*ssaV*). Future efforts should aim at the characterization of *proQ* mutant *Salmonella* in further *in vivo* models of infection and also consider the role of this RBP in breaching the barrier functions imposed by the intestinal microbiota.

10.1128/mBio.02504-18.9 FIG S9 *In vivo* phenotype of Δ*proQ Salmonella*. Streptomycin-pretreated C57BL/6 mice were infected (5 × 10^7^ CFU in total by gavage) with a 1:1 mixture of wild-type *Salmonella* (Str^r^) and the indicated isogenic mutant strains (Str^r^ Kan^r^). The Δ*ssaV* strain has a functional knockout of the T3SS of SPI-2 and was included as a positive control of an attenuated strain. Three days postinfection, the indicated organs were isolated and homogenized, and competitive indices were determined by selective plating. The data stem from five animals per group from two independently performed experiments (single data points are shown, and the geometric mean for a group is indicated by a black bar). Statistical significance was assessed by one-tailed Wilcoxon matched pair rank test, comparing the *proQ*-deficient strain to its coinfecting wild-type control strain. Statistically significant differences are indicated by asterisks as follows: *, *P = *0.0313; **, *P* values of ≤0.01. Despite the slight induction in basal *prc* expression in the Δ*proQ* background (Fig. S1a), we confidently assign the *in vivo* attenuation of the *proQ* mutant strain to the absence of this RBP, since *prc* overexpression would be expected to result in hyperreplication (86). Download FIG S9, PDF file, 1.3 MB. Copyright © 2019 Westermann et al. 2019 Westermann et al. This content is distributed under the terms of the Creative Commons Attribution 4.0 International license.

### Perspective.

We now know that deletion of any of the five global RBPs in *Salmonella* is associated with diverse virulence defects (Fig. 6b). In *Salmonella*, Hfq binds many virulence-related RNA transcripts (5, 7, 68) and hence, *hfq* deletion mutants show severe virulence defects, including dysregulated virulence factor expression and secretion, chemotaxis defects, and reduced motility (9, 10, 25). The translational control protein CsrA tunes expression of the master regulator of *Salmonella* invasion genes, HilD (8), and interacts with mRNAs for effectors of both major SPIs (5). Consequently, *Salmonella* mutants lacking CsrA are avirulent (69). Most recently, the cold shock proteins CspC and CspE were shown to interact with transcripts of PhoP regulon members in *Salmonella*, and a Δ*cspC* Δ*cspE* double deletion mutant is strongly attenuated during infection of both cell lines and mice (6). With respect to ProQ, the only pathogenesis-related process previously known to be affected was biofilm formation which was shown to be strongly reduced in E. coli Δ*proQ* (70). The results presented here for *Salmonella* ProQ further highlight the importance of RBP functions for bacterial virulence and should encourage more efforts to address the molecular mechanisms employed by global RBPs under infection settings.

## MATERIALS AND METHODS

### *Salmonella* strain construction and growth conditions.

Salmonella enterica serovar Typhimurium strain SL1344 (strain JVS-1574 [43]) and a constitutively GFP-expressing derivative thereof (JVS-3858 [28]) are considered wild-type. Chromosomal mutations were generated as previously described (71), the mutated alleles were subsequently transduced into one of the wild-type backgrounds (GFP negative or positive, respectively) using P22 phage (72), and the respective resistance cassettes were eliminated using the FLP helper plasmid pCP20 (71) at 42°C. For plasmid transformation, the respective *Salmonella* strains were electroporated with ∼10 ng of DNA. The complete lists of bacterial strains and plasmids used in this study are provided in Tables S1E and F in the supplemental material. Routinely, *Salmonella* strains were grown in liquid Lennox broth (LB) medium or on solid LB agar medium at 37°C. When appropriate, the liquid or solid medium was supplemented with 30 μg/ml chloramphenicol (Cm), 100 μg/ml ampicillin (Amp), 50 μg/ml kanamycin (Kan), or 0.02% (wt/vol) L-arabinose (final concentrations).

For *in vitro* assays reflecting defined virulence conditions, *Salmonella* overnight cultures were diluted 1:100 in 10 ml of LB and grown at 37°C with shaking at 220 rpm to an OD_600_ of 2.0 (ESP, i.e., a SPI-1-inducing condition [9, 64]). To reflect the conditions of the *Salmonella*-containing vacuole, 1 ml of an LB culture at an OD_600_ of 2.0 was pelleted, and the bacterial cells were washed two times with PBS and once with SPI-2-inducing minimal medium (42) and diluted 1:50 in 10 ml of this minimal medium. If not mentioned otherwise, cultures were grown at 37°C and 220 rpm until they reached an OD_600_ of 0.3.

### Mammalian cell culture techniques.

Human cervix carcinoma HeLa cells (ATCC CCL-2; for Fig. 2f) or HeLa-S3 cells (ATCC CCL-2.2; if not mentioned otherwise), and human monocytic THP-1 cells (ATCC TIB-202) were cultured as previously described (30). HeLa cells were passaged in DMEM (Gibco), and THP-1 cells were passaged in RPMI (Gibco), with each medium supplemented with 10% fetal calf serum (FCS) (Biochrom), 2 mM L-glutamine (Gibco), and 1 mM sodium pyruvate (Gibco) in T-75 flasks (Corning) in a 5% CO_2_, humidified atmosphere at 37°C, and routinely tested for mycoplasma contamination using the MycoAlert Mycoplasma Detection kit (Lonza). Two days prior to infection, 2 × 10^5^ cells were seeded in 2 ml of antibiotic-free medium (six-well format), resulting in a cell density of 1 × 10^6^ cells/well at the time of infection. To differentiate THP-1 cells into macrophages, monocytic THP-1 were seeded at 1 × 10^6^ cells/well (six-well format) 3 days prior to infection in medium supplemented with 50 ng/ml (final concentration) of phorbol 12-myristate 13-acetate (PMA) (Sigma), and the medium was replenished after 2 days.

### Infection assays.

Infection assays were conducted as previously described (30). To avoid loss of the *proQ* overexpression plasmid (and the respective empty control vector) during intracellular bacterial growth, we performed HeLa infection assays in the presence or absence of the respective selection marker (ampicillin) in the host cell medium (Fig. S1c). During the first 24 h of infection, no significant plasmid loss was observed, even in the absence of the antibiotic. Nevertheless, all future infection experiments were conducted in the presence of 1× ampicillin (=100 µg/ml final concentration) in the cell medium.

Briefly, overnight cultures of *Salmonella* were diluted 1:100 in fresh LB medium and grown aerobically to an OD_600_ of 2.0. Bacterial cells were harvested by centrifugation (2 min at 12,000 rpm, room temperature) and resuspended in host cell medium. Alternatively, to reduce THP-1 cytotoxicity (Fig. S2), overnight cultures were opsonized in mouse serum (Sigma) for 20 min at room temperature and used for infection. Either way, infection was carried out by adding the respective bacterial suspension directly to each well of seeded host cells. HeLa cells were infected at a multiplicity of infection (MOI) of 5, unless otherwise indicated, and THP-1 cells (both monocytes and macrophages) were infected at an MOI of 10. Immediately after addition of the bacteria, the plates were centrifuged for 10 min at 250 × *g* at room temperature, followed by 30-min incubation in 5% CO_2_, humidified atmosphere at 37°C. Thereafter, gentamicin and ampicillin were added to the medium at a final concentration of 50 µg/ml or 100 µg/ml, respectively. When infecting HeLa cells or differentiated THP-1 cells (i.e., adherent cells), the medium was replaced after a further 30-min incubation for host medium containing 10 μg/ml of gentamicin and 100 µg/ml of ampicillin and incubated for the remainder of the experiment. For the infection of nondifferentiated THP-1 cells (that grow in suspension), the medium was not replaced, and cells were kept in 50 µg/ml gentamicin and 100 µg/ml ampicillin until harvest. In each case, time zero was defined as the time when gentamicin was first added to the infected cells.

### Flow cytometry and fluorescence-activated cell sorting (FACS).

For flow cytometry-based analyses, infected cell cultures were washed twice with PBS, if necessary cells were detached from the bottom of the plate by trypsinization (HeLa cells) or scraping (differentiated THP-1 cells) and resuspended in host cell medium. Upon pelleting the cells (5 min at 250 × *g*, room temperature), they were resuspended in 4% (wt/vol) paraformaldehyde (PFA) and stored at 4°C until the analysis. To prepare the PFA-fixed cells for flow cytometry, the samples were centrifuged as described above, the pelleted cells were washed with PBS, centrifuged again, and resuspended in 250 µl of PBS per 1 × 10^6^ cells (i.e., one well of the six-well format). The cells were measured by flow cytometry using a BD Accuri C6 instrument (BD Biosciences), gating for GFP-positive cells in the FITC channel versus the autofluorescence in the PE channel as described elsewhere (29), and the data were analyzed using FlowJo software (Tree Star Inc.).

For the FACS-based enrichment of invaded (GFP-positive) HeLa-S3 cells, the infected cultures were washed once with PBS and trypsinized, and each three wells were pooled and transferred into 15-ml tubes and pelleted (5 min at 250 × *g*, 4°C). The supernatant was removed, the cell pellet was resuspended in 500 µl of RNA*later* (Qiagen), and stored at 4°C until sorting. Immediately prior to FACS, RNA*later*-fixed cell samples were diluted by adding each 10 ml of ice-cold PBS and pelleted (5 min at 500 × g, 4°C). The RNA*later* was removed by aspiration. The cell pellet was washed once in ice-cold PBS, resuspended in 250 µl of ice-cold PBS/1 × 10^6^ cells, and sorted using the FACSAria III device (BD Biosciences) at 4°C (cooling both the input tube holder and the collection tube rack) and at a medium flow rate as described previously (29). Typically ∼2 × 10^5^ cells of each fraction were collected and used for RNA isolation.

### CFU assays.

To assess plasmid loss during intracellular replication, infected HeLa cultures were solubilized with PBS containing 0.1% (vol/vol) Triton X-100 (Gibco) at 24 h p.i. The resulting cell lysates were serially diluted in PBS, plated onto LB or LB-Amp plates and incubated at 37°C overnight. The number of colony-forming units (CFU) recovered on the next day was compared to that obtained from the bacterial input solution used for infection. Biological triplicates were collected, each comprised of two technical replicates.

### RNA extraction and gDNA removal.

For Dual RNA-seq analysis, total bacterial and host RNA from infected cells was isolated using the *mir*Vana kit (Ambion). To this end, the fixed and sorted cells were pelleted (5 min, 1,000 × g, 4°C), lysed in 600 µl of the Lysis/Binding buffer of the *mir*Vana kit (Ambion), and the samples were further processed following the manufacturer’s instructions for total RNA isolation. For bacterial RNA-seq analyses, qRT-PCR experiments or Northern blots, total RNA was isolated using the TRIzol LS reagent (Invitrogen) according to the manufacturer’s recommendations. To remove contaminating genomic DNA, samples for (Dual) RNA-seq or qRT-PCR were further treated with 0.25 U of DNase I (Fermentas) per 1 µg of RNA for 45 min at 37°C.

### cDNA library preparation for (Dual) RNA-seq analyses.

To deplete ribosomal transcripts, RNA samples were treated with the Ribo-Zero “Epidemiology” (Dual RNA-seq) or “Bacteria” (*Salmonella*-only RNA-seq) kits (Illumina). Following the manufacturer’s instructions, ∼500 ng of total, DNase I-treated RNA was used as an input to the ribo-depletion procedure. rRNA-depleted RNA was precipitated in ethanol for 3 h at −20°C.

cDNA libraries for Illumina sequencing were generated by Vertis Biotechnologie AG, Freising-Weihenstephan, Germany. rRNA-free RNA samples were first sheared via ultrasound sonication (four 30-s pulses at 4°C) to generate on average 200- to 400-nt fragments. Fragments of <20 nt were removed using the Agencourt RNAClean XP kit (Beckman Coulter Genomics) and the Illumina TruSeq adapter was ligated to the 3′ ends of the remaining fragments. First-strand cDNA synthesis was performed using M-MLV reverse transcriptase (NEB) wherein the 3′ adapter served as a primer. The first-strand cDNA was purified, and the 5′ Illumina TruSeq sequencing adapter was ligated to the 3′ end of the antisense cDNA. The resulting cDNA was PCR amplified to about 10 to 20 ng/μl using a high fidelity DNA polymerase. The TruSeq barcode sequences were part of the 5′ and 3′ TruSeq sequencing adapters. The cDNA library was purified using the Agencourt AMPure XP kit (Beckman Coulter Genomics) and analyzed by capillary electrophoresis (Shimadzu MultiNA microchip).

### Illumina sequencing.

Generally, for sequencing, cDNA samples were pooled in approximately equimolar amounts. The cDNA pool was size fractionated in the size range of 200 to 600 bp using a differential cleanup with the Agencourt AMPure kit (Beckman Coulter Genomics). Aliquots of the cDNA pools were analyzed by capillary electrophoresis (Shimadzu MultiNA microchip). Sequencing was performed on a NextSeq 500 platform (Illumina) at Vertis Biotechnologie AG, Freising-Weihenstephan, Germany (single-end mode; 75 cycles).

### Computational methods to interpret RNA-seq data sets.

The adapter sequences as well as low-quality ends of the raw sequences were removed using *cutadapt* version 1.13 (73). Reads were mapped to the *Salmonella* reference sequence (NC_016810.1, NC_017718.1, NC_017719.1, and NC_017720.1) and for the Dual RNA-Seq samples additionally to the human reference genome (GRCh38.p10 from *GENCODE*) using *READemption*’s subcommand *align* (*READemption* version 0.4.3) (74) and *segemehl* 0.2.0. Coverage plots were generated with *READemption*’s subcommand *coverage*, and reads overlapping genomic features were quantified with the subcommand *gene_quanti* followed by expression level comparison with *edgeR* (version 3.22.1) (75). Sequencing coverages were visualized using the Integrated Genome Browser (IGB) (76) and are based on uniquely mapped reads normalized by the total number of aligned reads per organism. Host-pathogen pathway enrichment analyses in the Dual RNA-seq data set (Fig. 1a and 2a) were performed using gene set enrichment analysis (GSEA; version 2.1.0) (77) fed with the log_2_ fold changes reported by *edgeR*. *Salmonella* pathway enrichment analyses in the *in vitro* RNA-seq data set (Fig. 3e) were performed using the Gene Ontology (GO) knowledgebase and resources (78, 79).

### qRT-PCR.

qRT-PCR was performed with the Power SYBR Green RNA-to-CT1-Step kit (Applied Biosystems) according to the manufacturer’s instructions and a CFX96 Touch real-time PCR detection system (Bio-Rad). *gfp* mRNA (where applicable) or 5S rRNA served as *Salmonella* reference transcripts. Fold changes in expression were determined using the 2^−ΔΔCt^ method (80). Primer sequences are given in Table S1G, and their specificity had been confirmed using Primer-BLAST (NCBI).

### PCR to detect *Salmonella* flagellar phase variation.

To extract genomic DNA from wild-type *Salmonella*, 100 µl of an LB overnight culture of strain JVS-1574 was pelleted, and the bacteria were resuspended in 100 µl of water. The suspension was boiled for 5 min at 100°C, vortexed briefly, and centrifuged (5 min at 13,000 rpm, room temperature). To the supernatant, one volume of chloroform was added, the mixture was vortexed for 30 s and centrifuged for 10 min at 13,000 rpm and 4°C. One microliter of the aqueous phase served as the template for PCR amplification with *Taq* polymerase (NEB). Flanking PCR primers (JVO-14315 and JVO-14316) were designed to anneal outside of—and the internal primer (JVO-14313) to bind to—the invertible chromosomal region in front of the *fljB* promoter (according to reference 81). Denaturation was at 95°C for 30 s, annealing was at 60°C for 30 s, and elongation was for 1.5 min at 72°C. After 35 cycles, the PCR products were resolved on a 1.5% (wt/vol) agarose/TAE gel, stained with ethidium bromide, and visualized using a UV transilluminator (Intas Science Imaging).

### Northern blotting.

Each 5 μg of total *Salmonella* RNA prepared with TRIzol LS reagent (Invitrogen) was loaded per lane and separated in 6% (vol/vol) polyacrylamide–7 M urea gels. Blotting was performed as previously described (30). After the transfer onto Hybond XL membranes (Amersham), RNA was cross-linked with UV light and hybridized at 42°C with gene-specific ^32^P-end-labeled DNA oligonucleotides (see Table S1G for probe sequences) in Hybri-Quick buffer (Carl Roth AG). After exposure, the screens were read out on a Typhoon FLA 7000 phosphorimager (GE Healthcare).

### Western blotting.

Immunoblotting was performed as previously described (30). Briefly, samples from *Salmonella in vitro* cultures were taken corresponding to an OD_600_ of 0.4 and centrifuged for 4 min at 16,100 × *g* at 4°C, and pellets were resuspended in sample loading buffer to a final concentration of 0.01 OD per μl. After denaturation for 5 min at 95°C, 0.05-OD equivalents of the sample were separated via SDS-PAGE (10% [wt/vol] PAA). Gel-fractionated proteins were blotted for 90 min (0.2 mA per cm^2^; 4°C) in a semidry blotter (Peqlab) onto a PVDF membrane (Perkin Elmer) in transfer buffer (25 mM Tris base, 190 mM glycine, 20% [vol/vol] methanol). Blocking was for 1 h at room temperature in 10% (wt/vol) dry milk/TBST20. Appropriate primary antibodies (Table S1H) were hybridized at 4°C overnight, and after three 10-min washes with TBST20, secondary antibodies (Table S1H) were hybridized for 1 h at room temperature. After three additional washing steps for each 10 min in TBST20, blots were developed using Western lightning solution (Perkin Elmer) in a Fuji LAS-4000 imager (GE Healthcare). When needed, quantification of the signal intensities of protein bands was performed using ImageJ (82).

For Western blotting of human proteins, infected HeLa cultures were trypsinized and sorted, and each ∼2 × 10^5^ GFP-positive or -negative cells were collected, pelleted, and lysed in 50 µl of sample loading buffer. Alternatively, ∼2 × 10^6^ cells (i.e., one well; six-well format) of an infected culture were directly lysed in 500 µl of sample loading buffer. In each case, the lysates were boiled for 5 min at 95°C, and each 20 -μl lysate was loaded per lane of a 10% (wt/vol) PAA gel for SDS-PAGE as described above. After blotting and blocking (as described above), the membrane was probed with the respective primary antibody at 4°C overnight, and after washing (as described above), the membrane was probed with the secondary antibody for 1 h at room temperature (see Table S1H for a list of antibodies). Detection of tubulin was as described above. To detect cFOS, the SuperSignal West Femto maximum sensitivity substrate (Thermo Scientific) was used.

### sRNA pulse expression.

The plasmids pBAD-ctrl. (pKP-8), pBAD-STnc540-L (pAW-16), or pBAD-STnc540-S (pAW-17) (Table S1F) were transformed into the Δ*STnc540* background. Pulse expression of the respective isoform of STnc540 was induced from the pBAD promoter by the addition of 0.02% (wt/vol) L-arabinose (Sigma) to the respective cultures in minimal SPI-2-inducing medium at an OD_600_ of 0.3. Ten minutes after the induction, total RNA was extracted using TRIzol LS reagent (Invitrogen), treated with DNase I, and analyzed by RNA-seq.

### GFP reporter assays.

To monitor *ssaG* or *mgtC* promoter induction and translational MgtC and MgtB activity in real time, GFP reporters were reused (P*_ssaG_*_::_*_gfp_* [30]) or newly constructed by fusing truncations of the 5′ part of the *mgt* operon to the GFP open reading frame as shown in Fig. S8a. To this end, the respective regions were PCR amplified from *Salmonella* genomic DNA and inserted into the plasmid pAS-93 via AatII/NheI sites as previously described (83). The resulting reporter plasmids (pYC-104, pYC-101, pAW-37, and pAW-38) or a constitutive GFP expression plasmid (pXG-1) were cotransformed with either pZE-ctrl. (pJV-300), pZE-STnc540-L (pAW-24), pZE-STnc540-S (pAW-25), or pZE12-ProQ into the Δ*STnc540*, Δ*proQ*, or Δ*STnc540* Δ*proQ* strain background. The resulting strains were grown overnight in LB-Amp/Cm, then diluted 1:100, and further grown in the same medium to an OD_600_ of 2.0. A volume of 1 ml of the cultures was pelleted and the collected cells were shifted to SPI-2-inducing medium (defined as time point zero). For the P*_ssaG_* reporter assay (Fig. 2g), the respective strains were grown to defined optical densities in 96-well plates (Nunc Microwell 96F; Thermo Scientific) at 37°C (with shaking), and the GFP signal intensity was recorded using the Infinite F200 PRO plate reader (Tecan). For the temporal measurement of the activity of *mgt* reporters (Fig. 5e and Fig. S8), the strains were grown under the same conditions for 24 h, and GFP levels were monitored in 10-min intervals (Infinite F200 PRO plate reader; Tecan). In parallel, the OD_595_ was recorded, and the respective values were used to normalize the GFP signals to cell density.

### *In silico* prediction of sRNA-mRNA interactions.

RNA duplex formation between STnc540 and the *mgt* operon (Fig. S6d) was predicted using the *RNAhybrid* program (49) with default settings.

### Animal infection experiments.

Animal housing and experimentation were approved by the Kantonales Veterinäramt Zürich, Switzerland (licenses 223/2010 and 222/2013). Wild-type C57BL/6 mice were kept under specific-pathogen-free (SPF) barrier conditions in individually ventilated cages at the EPIC facility, ETH Zürich. *Salmonella* infections were performed according to previously published protocols (84). Briefly, 8- to 12-week-old mice were pretreated with 25 mg of streptomycin by gavage 24 h before the infection. The indicated *Salmonella* strains were grown in LB/0.3 M NaCl with appropriate antibiotics at 37°C for 12 h and subcultured to late log phase for 4 h in the same medium lacking antibiotics. The bacteria were washed in PBS and mixed in a 1:1 ratio, and a total of 5 × 10^7^ CFU were inoculated by gavage. *Salmonella* loads in the indicated organs were monitored by plating serial dilutions of homogenized cecal content or tissue on MacConkey agar supplemented with appropriate antibiotics. The competitive index (CI) was calculated as the ratio of competitor population sizes in the infected organ corrected for the initial ratio in the inoculum. A CI of <1 means that the indicated strain loses against the wild-type strain (JVS-1574).

### Data availability.

All RNA-seq data discussed in this publication have been deposited in NCBI’s Gene Expression Omnibus and are accessible through GEO Series accession number GSE117256. The workflows of the different analyses represented as Shell scripts are deposited at Zenodo (https://doi.org/10.5281/zenodo.1311214).
