# Supplementary figures and images for: The Major RNA-Binding Protein ProQ Impacts Virulence Gene Expression in Salmonella enterica Serovar Typhimurium
Source: mBio. 2019 Jan 2;10(1):e02504-18. doi: 10.1128/mBio.02504-18 (PMC6315103; doi:10.1128/mBio.02504-18)

# Supplementary Figure S1

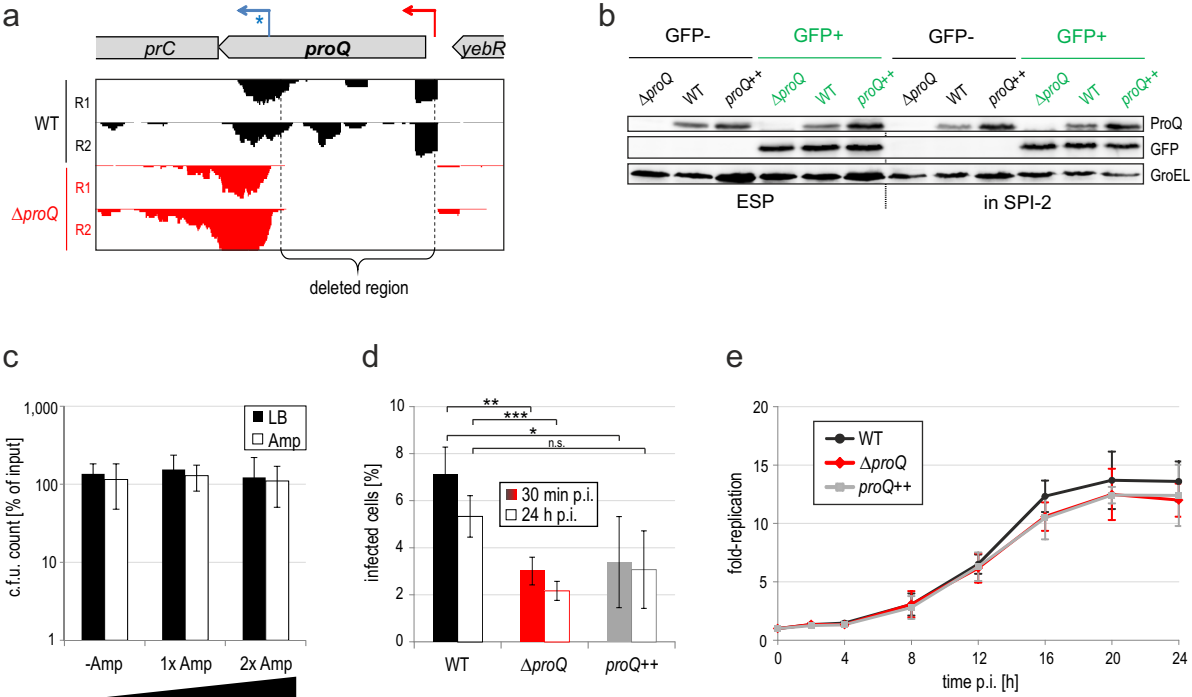

Supplement: FIG S1 [file mbo006184234sf1.pdf]

Supplementary Figure S2

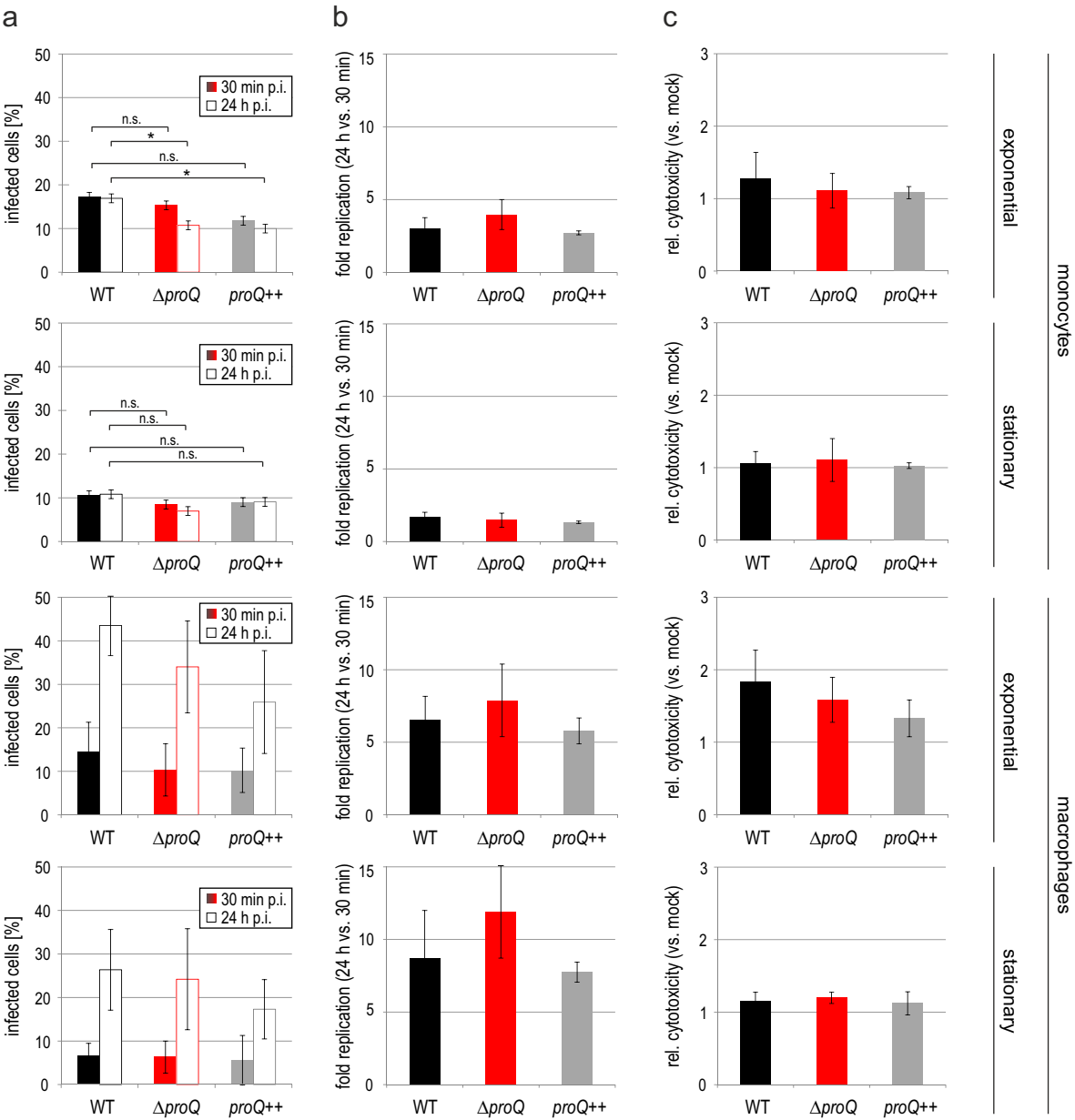

Supplement: FIG S2 [file mbo006184234sf2.pdf]

# Supplementary Figure S3

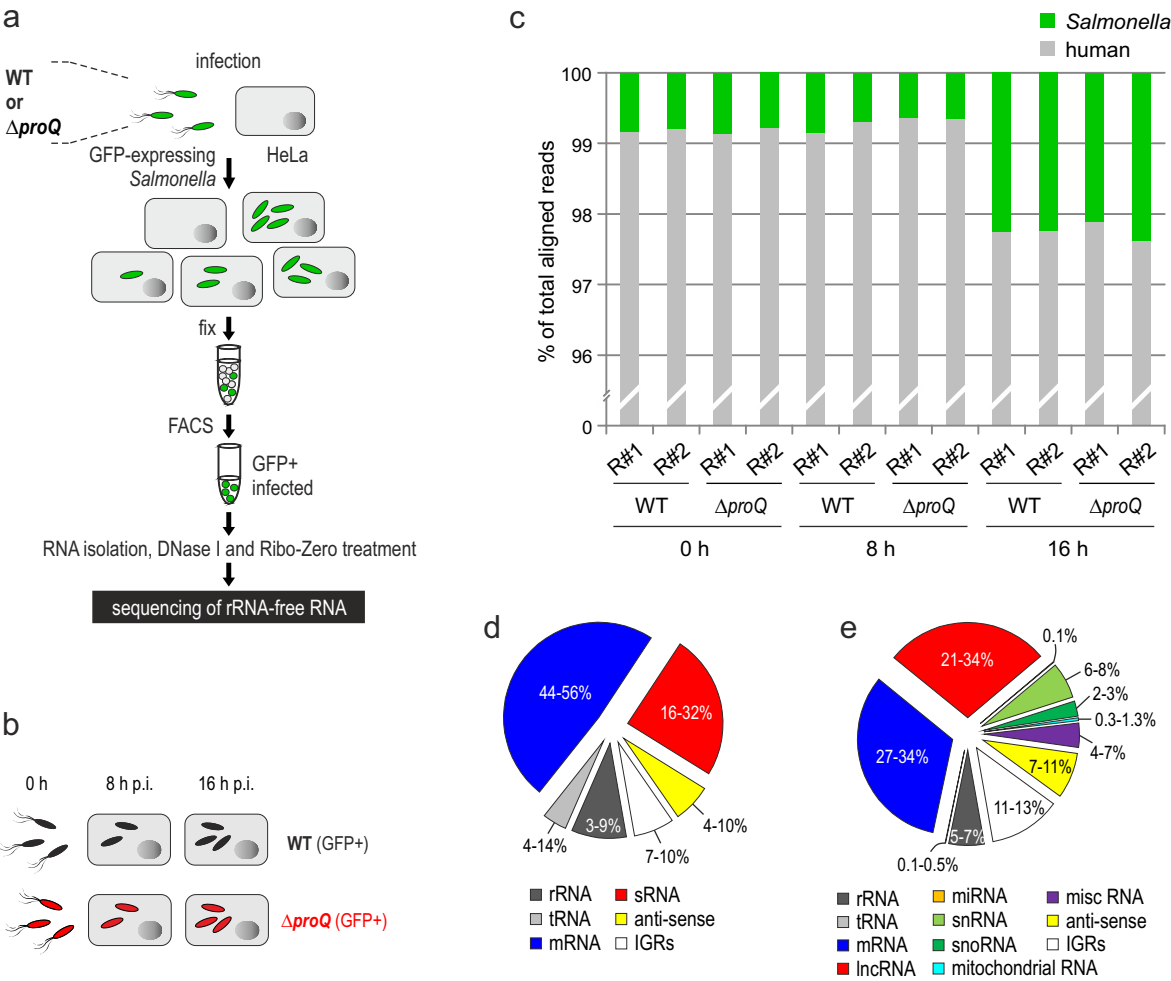

Supplement: FIG S3 [file mbo006184234sf3.pdf]

# Supplementary Figure S4

a

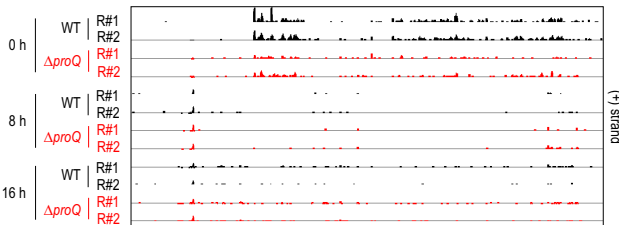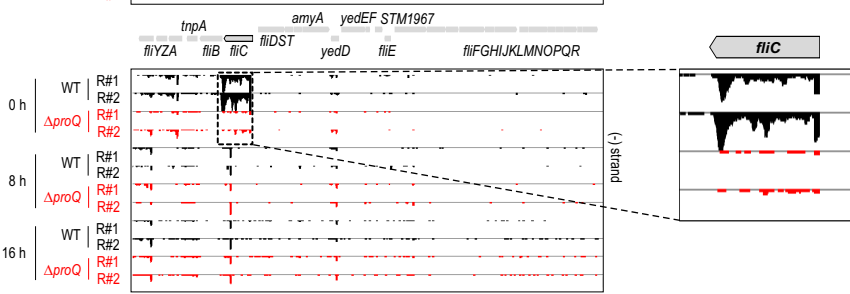

b

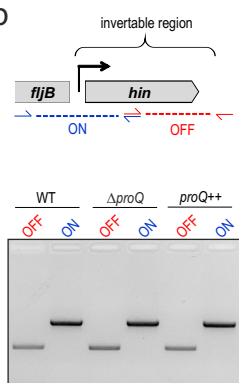

c

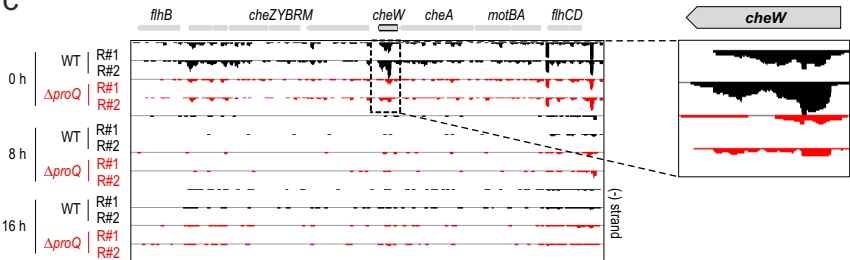

d

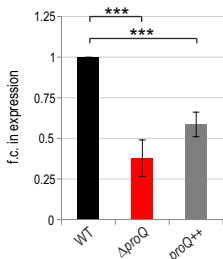

e

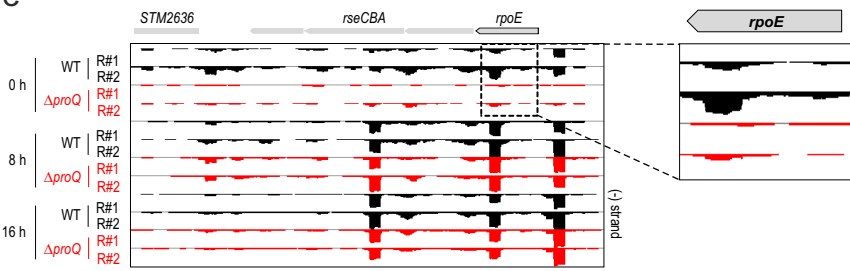

f

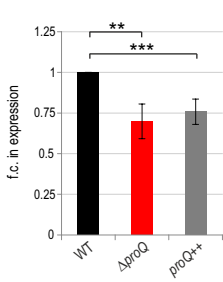

g

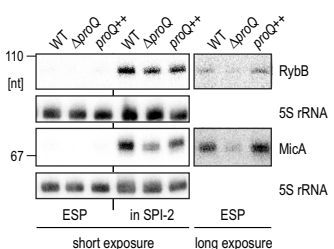

Supplement: FIG S4 [file mbo006184234sf4.pdf]

# Supplementary Figure S5

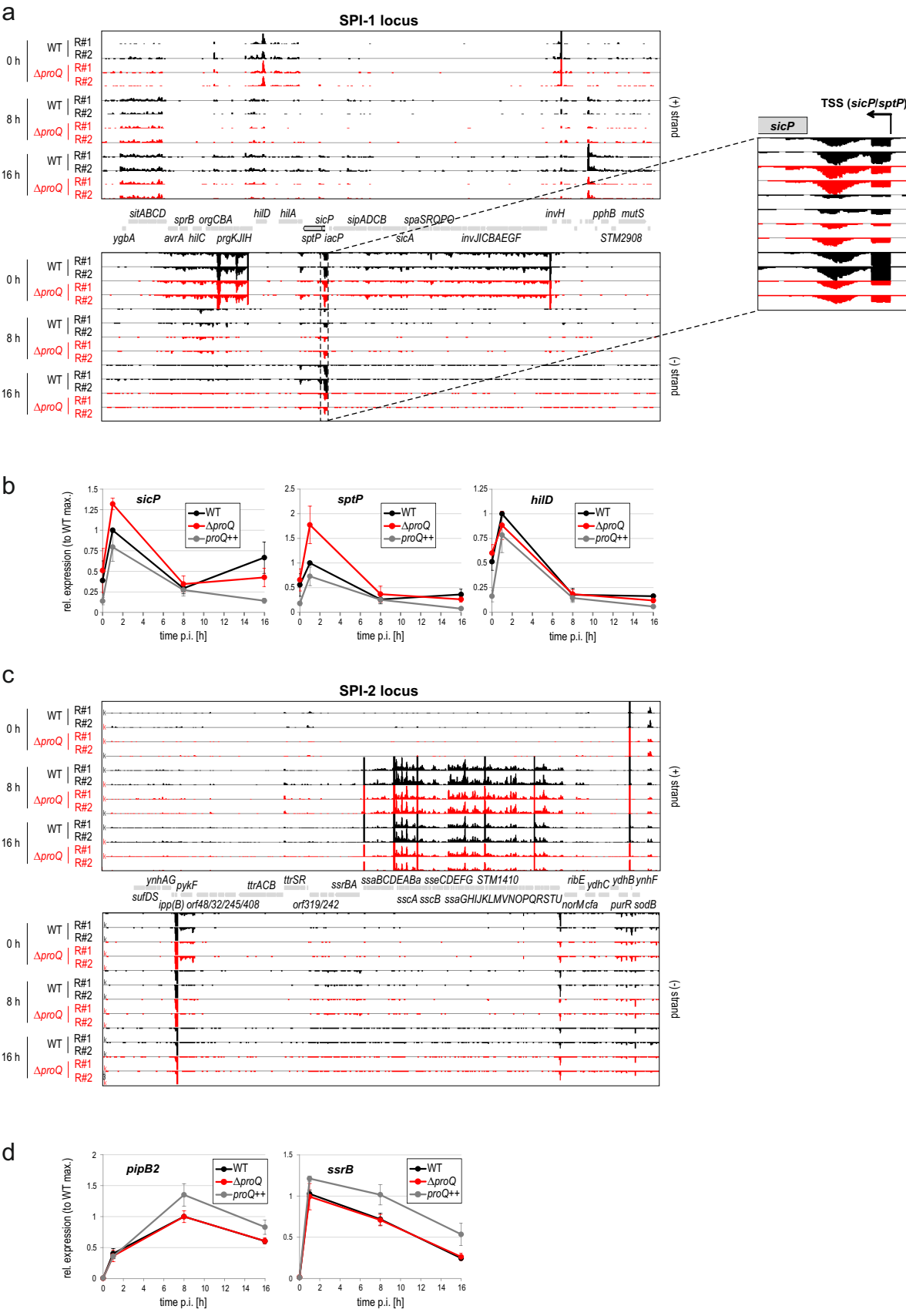

Supplement: FIG S5 [file mbo006184234sf5.pdf]

# Supplementary Figure S6

a

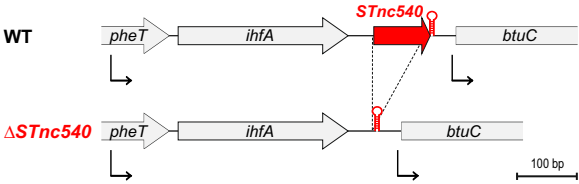

b

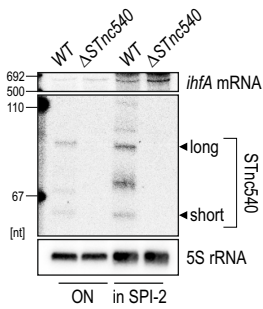

c

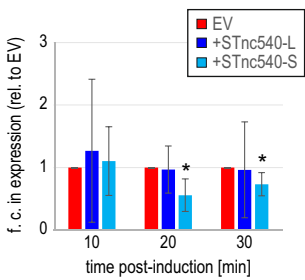

d

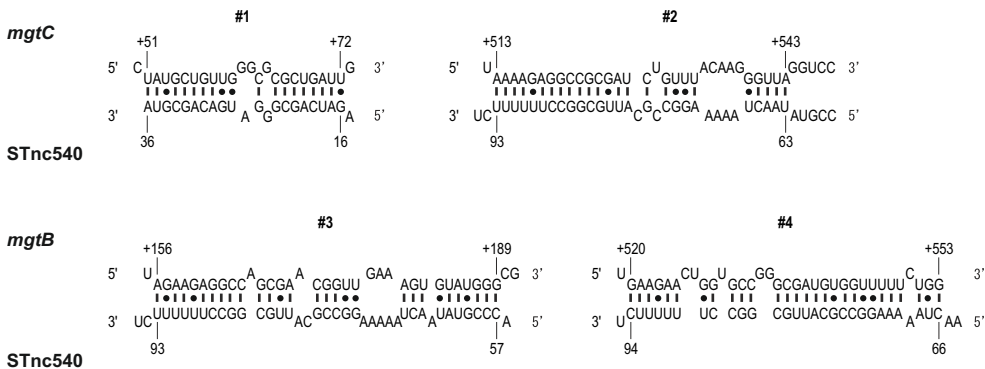

Supplement: FIG S6 [file mbo006184234sf6.pdf]

Supplementary Figure S7

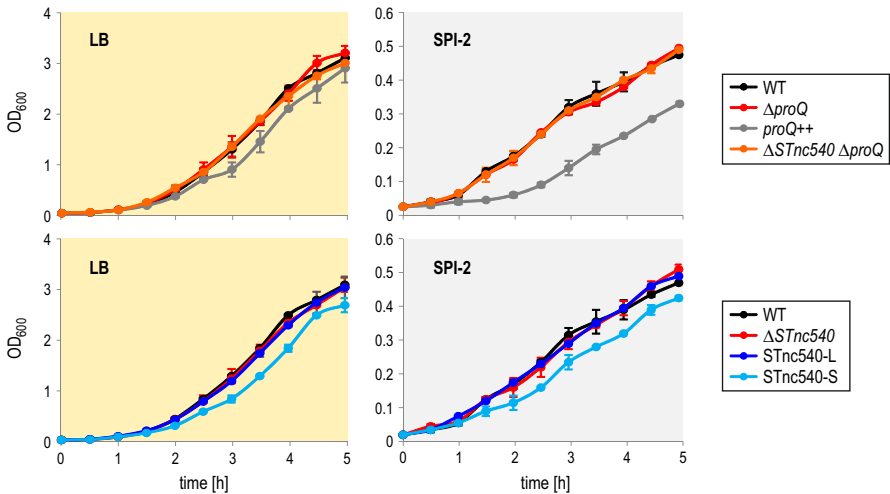

Supplement: FIG S7 [file mbo006184234sf7.pdf]

# Supplementary Figure S8

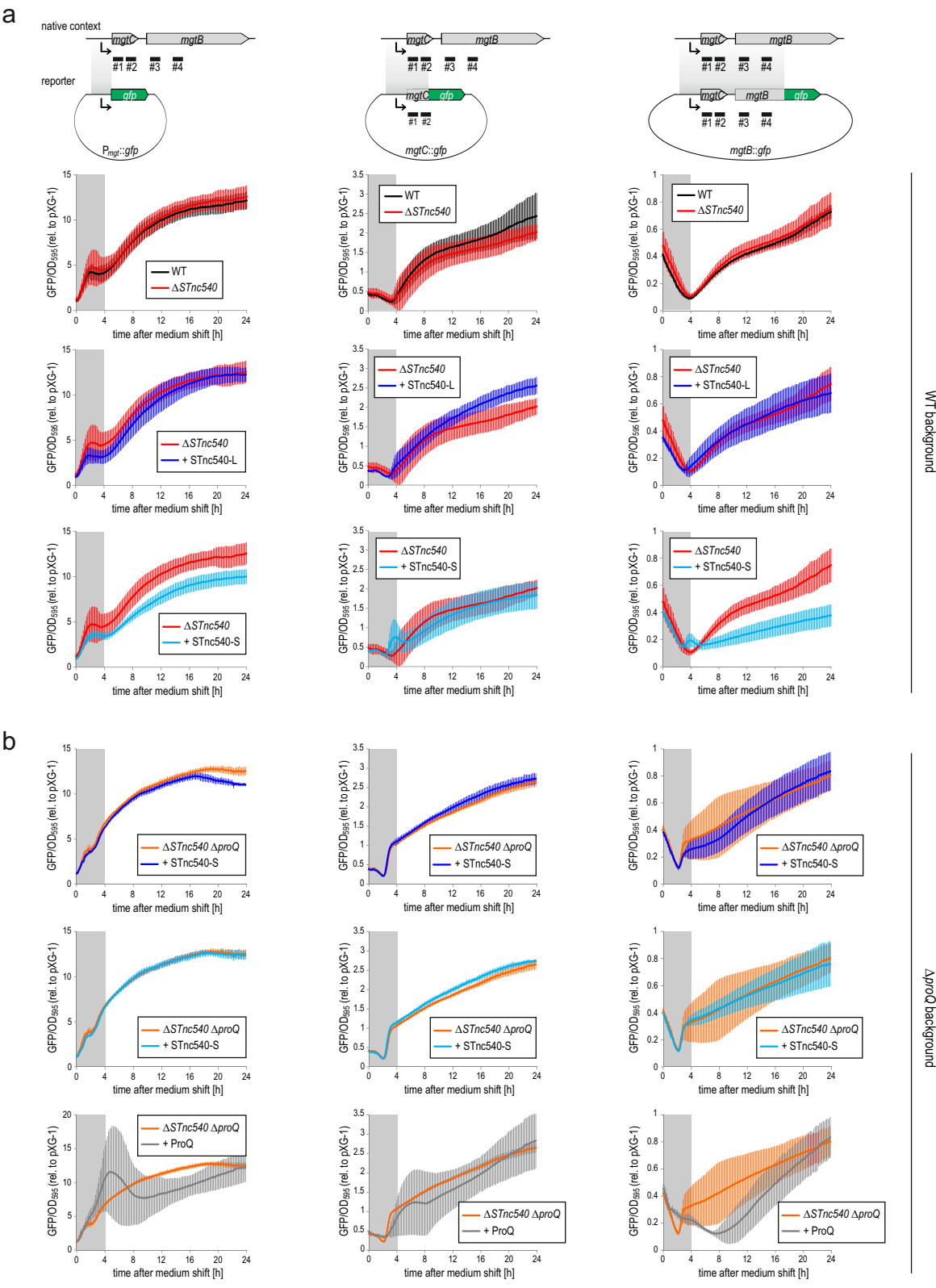

Supplement: FIG S8 [file mbo006184234sf8.pdf]

Supplementary Figure S9

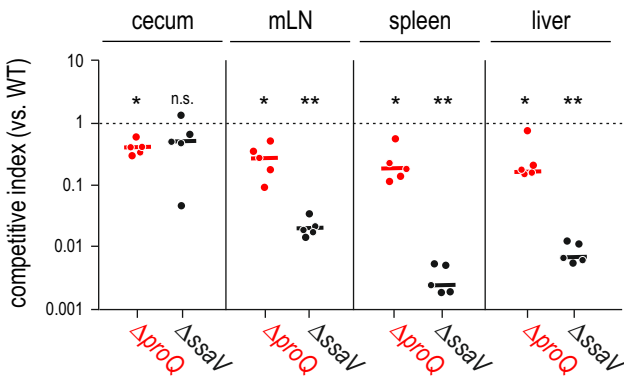

Supplement: FIG S9 [file mbo006184234sf9.pdf]
